# Kir2.1-Nav1.5 Channel Complexes Are Differently Regulated than Kir2.1 and Nav1.5 Channels Alone

**DOI:** 10.3389/fphys.2017.00903

**Published:** 2017-11-14

**Authors:** Raquel G. Utrilla, Paloma Nieto-Marín, Silvia Alfayate, David Tinaquero, Marcos Matamoros, Marta Pérez-Hernández, Sandra Sacristán, Lorena Ondo, Raquel de Andrés, F. Javier Díez-Guerra, Juan Tamargo, Eva Delpón, Ricardo Caballero

**Affiliations:** ^1^Department of Pharmacology, School of Medicine, Instituto de Investigación Sanitaria Gregorio Marañón, Universidad Complutense, Madrid, Spain; ^2^CIBERCV, Madrid, Spain; ^3^Departamento de Biología Molecular and Centro de Biología Molecular “Severo Ochoa” (UAM-CSIC), Universidad Autónoma de Madrid, Madrid, Spain

**Keywords:** Nav1.5, Kir2.1, heart, ion channel biology, patch-clamp

## Abstract

Cardiac Kir2.1 and Nav1.5 channels generate the inward rectifier K^+^ (I_K1_) and the Na^+^ (I_Na_) currents, respectively. There is a mutual interplay between the ventricular I_Na_ and I_K1_ densities, because Nav1.5 and Kir2.1 channels exhibit positive reciprocal modulation. Here we compared some of the biological properties of Nav1.5 and Kir2.1 channels when they are expressed together or separately to get further insights regarding their putative interaction. First we demonstrated by proximity ligation assays (PLAs) that in the membrane of ventricular myocytes Nav1.5 and Kir2.1 proteins are in close proximity to each other (<40 nm apart). Furthermore, intracellular dialysis with anti-Nav1.5 and anti-Kir2.1 antibodies suggested that these channels form complexes. Patch-clamp experiments in heterologous transfection systems demonstrated that the inhibition of the Ca^2+^/calmodulin-dependent protein kinase II (CaMKII) decreased the I_Na_ and the I_K1_ generated by Nav1.5 and Kir2.1 channels when they were coexpressed, but not the I_K1_ generated by Kir2.1 channels alone, suggesting that complexes, but not Kir2.1 channels, are a substrate of CaMKII. Furthermore, inhibition of CaMKII precluded the interaction between Nav1.5 and Kir2.1 channels. Inhibition of 14-3-3 proteins did not modify the I_Na_ and I_K1_ densities generated by each channel separately, whereas it decreased the I_Na_ and I_K1_ generated when they were coexpressed. However, inhibition of 14-3-3 proteins did not abolish the Nav1.5-Kir2.1 interaction. Inhibition of dynamin-dependent endocytosis reduced the internalization of Kir2.1 but not of Nav1.5 or Kir2.1-Nav1.5 complexes. Inhibition of cytoskeleton-dependent vesicular trafficking via the dynein/dynactin motor increased the I_K1_, but reduced the I_Na_, thus suggesting that the dynein/dynactin motor is preferentially involved in the backward and forward traffic of Kir2.1 and Nav1.5, respectively. Conversely, the dynein/dynactin motor participated in the forward movement of Kir2.1-Nav1.5 complexes. Ubiquitination by Nedd4-2 ubiquitin-protein ligase promoted the Nav1.5 degradation by the proteasome, but not that of Kir2.1 channels. Importantly, the Kir2.1-Nav1.5 complexes were degraded following this route as demonstrated by the overexpression of Nedd4-2 and the inhibition of the proteasome with MG132. These results suggested that Kir2.1 and Nav1.5 channels closely interact with each other leading to the formation of a pool of complexed channels whose biology is similar to that of the Nav1.5 channels.

## Introduction

Kir2.1 channels generate the inward rectifier K^+^ current (I_K1_) that plays a key role in the control of the resting membrane potential and the duration of the late-phase of repolarization in human cardiac cells (Anumonwo and Lopatin, 2010; de Boer et al., 2010). On the other hand, Nav1.5 channels carry the inward Na^+^ current (I_Na_), which determines the Na^+^ influx that depolarizes the membrane potential during the upstroke of the action potential (Abriel, 2010; Wilde and Brugada, 2011). It has been recently demonstrated that there is a positive reciprocal modulation between Kir2.1 and Nav1.5 channels, in such a way that an increase in Kir2.1 channels expression leads to the increase of the I_Na_ density in cardiac ventricular myocytes and *vice versa* (Milstein et al., 2012; Matamoros et al., 2016). The reciprocal modulation is mediated by the binding of both channel types to α1-syntrophin, a scaffolding protein containing a PDZ domain (Matamoros et al., 2016). Indeed Nav1.5 channels interact with α1-syntrophin via two different PDZ-binding domains, one the canonical, constituted by the three last C-terminal residues (SIV) and another “PDZ-like” domain, determined by the presence of Ser20 and located “internally” at the N-terminus of the channel (Matamoros et al., 2016). Conversely, Kir2.1 channels exhibit a unique α1-syntrophin binding site within its C-terminal PDZ-binding domain (Matamoros et al., 2016), suggesting that Nav1.5, but not Kir2.1, could bind two molecules of α1-syntrophin at a time (Matamoros et al., 2016). These results suggested that at least some Nav1.5 and Kir2.1 channels form a multiprotein complex in which they interact directly or indirectly. The aims of the present work are to explore whether these complexes, if any, are formed just at the plasma membrane or at early stages of the protein assembly, as well as to characterize some of their biological properties (such as their anterograde or retrograde trafficking routes). The existence of such complexes would allow a dynamic and delicate control of the expression of these cardiac ion channels whose balanced function is critical for an adequate control of the excitability and cardiac impulse propagation. The results obtained demonstrate that at least a pool of Kir2.1 and Nav1.5 channels are in close proximity and interact at the membrane of cardiac cells forming complexes with anterograde and retrograde trafficking routes similar to those of the Nav1.5 channels alone.

## Methods

### Kir2.x and Nav1.5 constructs and Chinese Hamster Ovary (CHO) cell transfection

Human Kir2.1 (kindly provided by Dr. José Jalife; University of Michigan, USA) was subcloned into pcDNA3.1 plasmid (Invitrogen, USA). Human cardiac Nav1.5 (hH1) and Navβ1 cDNA subcloned in pCGI vector were kindly gifted by Dr. Connie R. Bezzina (University of Amsterdam, The Netherlands). CHO cells were cultured as previously described (Caballero et al., 2010b, 2017; Núñez et al., 2013; Matamoros et al., 2016) and transiently transfected with the cDNA encoding Nav1.5 channels (1.6 μg) and hNavβ1 (1.6 μg; Nav1.5-β) alone or together with Kir2.1 (1.6 μg) plus the cDNA encoding the CD8 antigen (0.5 μg) by using FUGENE XtremeGENE (Roche Diagnostics, Switzerland) following manufacturer instructions. Forty-eight hours after transfection, cells were incubated with polystyrene microbeads precoated with anti-CD8 antibody (Dynabeads M450; Life Technologies, USA). Most of the cells that were beaded also had channel expression. Coexpression of Nav1.5 and Kir2.1 channels was always tested electrophysiologically and only cells coexpressing both channels were patched.

Human Nedd4-2 (KIAA0439), p.K44A dynamin-2 mutant, and p.R56,60A 14-3-3 (14-3-3DN) mutant cDNAs subcloned in pcDNA3.1 were kindly gifted by Prof. Dr. Hugues Abriel (University of Bern, Switzerland), Dr. Federico Sesti (Rutgers University, USA), and Dr. Isabelle Baro (INSERM, France), respectively. In some experiments, Kir2.1 and Nav1.5 channels were cotransfected with the p50 subunit of the dynein/dynactin complex (1.6 μg, Origene, USA), p.K44A dynamin-2 (1.6 μg), 14-3-3DN (1.6 μg), or Nedd4-2 (1.6 μg).

### Patch-clamping

Kir2.1 (I_Kir2.1_) and Nav1.5 (I_Nav1.5_) currents were recorded at room temperature (21-23°C) using the whole-cell patch-clamp technique using an Axopatch-200B amplifier (Molecular Devices, USA) (Caballero et al., 2010a,b, 2017; Núñez et al., 2013; Matamoros et al., 2016). Recording pipettes were pulled from 1.0 mm o.d. borosilicate capillary tubes (GD1, Narishige Co., Ltd, Japan) using a programmable patch micropipette puller (Model P-2000 Brown-Flaming, Sutter Instruments Co., USA) and were heat-polished with a microforge (Model MF-83, Narishige). Micropipette resistance was kept below 3.5 and 1.5 MΩ for I_Kir2.1_ and I_Nav1.5_ recordings, respectively, when filled with the internal solution and immersed in the external solution. In all experiments, series resistance was compensated manually by using the series resistance compensation unit of the Axopatch amplifier, and ≥80% compensation was achieved. Uncompensated access resistance and CHO cell capacitance were 1.6 ± 0.3 MΩ and 12.8 ± 0.2 pF (*n* = 663), respectively. Peak I_Kir2.1_ and I_Nav1.5_ amplitudes were −2.3 ± 0.1 and −2.9 ± 0.2 nA, respectively. Therefore, under our experimental conditions no significant voltage errors (<5 mV) due to series resistance were expected with the micropipettes used.

To record I_Kir2.1_ and I_Nav1.5_, CHO cells were perfused with an external solution containing (mM): NaCl 136, KCl 4, CaCl_2_ 1.8, MgCl_2_ 1, HEPES 10, and glucose 10 (pH 7.4 with NaOH). Recording pipettes were filled with an internal solution containing (mM): K-aspartate 80, KCl 42, KH_2_PO_4_ 10, MgATP 5, phosphocreatine 3, HEPES 5, and EGTA 5 (pH 7.2 with KOH) to record I_Kir2.1_ (liquid junction potential-LJP = −13.2 mV) or NaF 10, CsF 110, CsCl 20, HEPES 10, and EGTA 10 (pH 7.35 with CsOH) to record I_Nav1.5_. I_Kir2.1_ current-voltage (I-V) curves were corrected according to the calculated LJP between the pipette and external solution (Caballero et al., 2010b, 2017; Matamoros et al., 2016). To minimize the contribution of time-dependent shifts of channel availability during sodium current recordings, all data were collected 20 min after establishing the whole-cell configuration. Under these conditions current amplitudes and voltage dependence of activation and inactivation were stable during the time of recordings (Matamoros et al., 2016; Caballero et al., 2017).

In some experiments, I_Kir2.1_ and I_Nav1.5_ were recorded in transiently transfected CHO cells dialyzed with anti-Kir2.1 or anti-Nav1.5 antibodies added to the internal solution (Archer et al., 1998). For the experiments conducted to determine the effect of the presence of both Nav1.5 and Kir2.1 on channel internalization kinetics, I_Nav1.5_ and I_Kir2.1_ were recorded at different time points after addition of 50 ng/mL brefeldin-A (BFA) to the culture medium (Rougier et al., 2005). In some experiments, cells were incubated with the Ca^2+^/calmodulin-dependent protein kinase II (CaMKII) inhibitor KN93 (1 μM, 24 h; Wagner et al., 2006) or with the proteasome inhibitor MG132 (20 μM, 2 h; Shy et al., 2014). In another group of patch-clamp experiments the CaMKII inhibitor autocamtide-2-related inhibitory peptide (AIP) was added to the internal solution (1 nM) that dialyzes the cells.

### Pulse protocols and analysis

The protocol to record I_Nav1.5_ consisted of 50-ms pulses in 5 mV increments from −120 mV to potentials between −80 and +50 mV. I_Nav1.5_ amplitude was measured at the peak of the current traces. For I_Kir2.1_, the protocol to obtain I-V curves consisted of 250-ms pulses in 10 mV increments from −60 mV to potentials between −120 and +20 mV (Caballero et al., 2010b, 2017; Matamoros et al., 2016). I_Kir2.1_ amplitude was measured at the end of the pulse. I_Kir2.1_ and I_Nav1.5_ recordings were sampled at 4 and 50 kHz, respectively, filtered at half the sampling frequency, and stored on the hard disk of a computer for subsequent analysis. Data were analyzed using pCLAMP software (Molecular Devices). In each experiment, current amplitudes were normalized to membrane capacitance to obtain current densities.

### Rat ventricular myocyte isolation

Single ventricular myocytes were isolated from hearts of male Sprague–Dawley rats (225–250 g) by enzymatic dissociation with collagenase type II (Worthington) following previously described methods (Matamoros et al., 2016; Caballero et al., 2017). Rats were heparinized (1.000 U/kg i.p.) and anesthetized with sodium pentobarbital (50 mg/kg i.p.). Animal studies were approved by the University Committee on the Use and Care of animals at the Complutense University and conformed to the Guidelines from Directive 2010/63/EU of the European Parliament on the protection of animals used for scientific purposes.

### Proximity ligation assay

The Duolink® (Sigma, USA) proximity ligation assay (PLA) was conducted by using previously described methods (Söderberg et al., 2006; Shy et al., 2014) in non-permeabilized rat ventricular myocytes. This assay allows for the *in situ* detection of proteins that are located in close proximity to each other (<40 nm apart). Experiments were performed according to the manufacturer's recommendations and using all incubation buffers without detergents in order to avoid cell membrane permeabilization. Isolated ventricular myocytes were fixed and incubated in blocking solution and then, incubated with primary antibodies as specified in previously described methods (Núñez et al., 2013). Subsequent incubation steps were performed at 37°C and the appropriate washing buffers were used after each incubation, as specified in the manufacturer's protocol. After washing three times in phosphate buffered saline (PBS) to remove the primary antibody, Duolink secondary antibodies, conjugated to PLA probes, were added to the myocytes for a 1 h incubation. These PLA probes consist of oligonucleotides which were subsequently joined together in a circle after addition of ligation solution (containing ligase enzyme) and incubation for 30 min. Next, rolling circle amplification of this circular template was achieved by addition of amplification solution, containing polymerase and fluorescently labeled oligonucleotides which hybridized to the circular template during 100 min of incubation. Samples were then mounted with Duolink *in situ* mounting medium with 4′,6-diamidino-2-phenylindole (DAPI) and viewed with a confocal microscope. Cell images and fluorescent signals were acquired on a Leica TCS-SP5 AOBS confocal microscope (Mannheim, Germany) with a 40x and 63x oil objectives, using a tetramethylrhodamine (TRITC) filter to visualize fluorescently labeled oligonucleotides (λ_ex_ = 554 nm; λ_em_ = 579 nm). Laser lines at 561 and 358 nm for excitation of fluorescently labeled oligonucleotides and DAPI were provided by a diode-pumped solid-state laser and a UV laser, respectively. Samples without primary antibodies were used as negative controls. Images were analyzed with ImageJ software (National Institutes of Health, USA) assigning green color to images taken under TRITC channel.

### Cell imaging

Cell surface labeling of Kir2.1 channels cotransfected or not with Nav1.5 in Human Embryonic Kidney-293 (HEK-293) cells was performed by confocal microscopy (Núñez et al., 2013; Confocal Microscope Zeiss LSM710 with objective 63X/1.4 Plan-Apochromat Oil DIC M27). Kir2.1 channels were fused to the circularly permuted Venus 173 (Kir2.1-CpVenus) fluorescent protein and images were acquired by exciting Venus at 500 nm and monitoring its emission at 535/22 nm. The capture conditions of the confocal microscope were optimized to obtain very thin optical sections (<300 nm, <1 Airy unit) as stated by the Nyquist sampling criterium (Pawley, 2006). ImageJ software was used to process the images and measure signal intensity (Núñez et al., 2013).

### Co-immunoprecipitation and western blot analysis

Co-immunoprecipitation was performed in CHO cells as previously described (Matamoros et al., 2016). Forty-eight hours after transfection with the plasmids encoding Kir2.1-CpVenus and/or Nav1.5 channel, CHO cells were washed with cold PBS and lysed in RIPA buffer to extract proteins and, thereafter, incubated for 30 min at 4°C. Precleared supernatant was incubated with 2 μg anti-Kir2.1 (N112B/14; Neuromab), 4 μg anti-CaMKIIδ (A010-56AP; Badrilla) or 3.2 μg anti-Nav1.5 (S0819, Sigma) antibodies for 3 h at 4°C, after which 20 μl of protein A/G-Sepharose beads (Santa Cruz Biotechnology Inc, USA) were added overnight at 4°C in rotation. Protein-antibody complexes were centrifuged and washed four times with cold PBS buffer. After the last centrifugation, 30 μl of loading dye with β-mercaptoethanol were added to the protein–antibody complexes and lysates and supernatants were prepared adding 20 μl of protein plus 30 μl of loading dye with β-mercaptoethanol. Thereafter, all samples were heated at 95°C for 5 min and then separated by SDS/PAGE and immunoblotted following described methods (Matamoros et al., 2016; Caballero et al., 2017). Briefly, samples were run on 10% Mini-PROTEAN® TGX™ gels (Bio-Rad, USA) and transferred to nitrocellulose membranes (Bio-Rad). Non-specific binding sites were blocked with 5% non-fat dry milk in PBS with Tween-20 (0.5%) for 1 h at room temperature. Membranes were then incubated with anti-GFP or anti-CaMKIIδ specific primary antibodies diluted in 5% non-fat dry milk overnight at 4°C. After washing, membranes were incubated with peroxidase-conjugated secondary antibodies or HRP-protein A. Antigen complexes were detected using enhanced chemiluminescence (Supersignal West Femto, Thermofisher, UK) and visualized using the Chemidoc MP System and Image Lab 5.2.1. software (Bio-Rad). Original blots are shown in Supplemental Figures 3–5.

### Antibodies

In the PLA, mouse monoclonal anti-Nav1.5 (1:50; ab62388, Abcam, UK) and rabbit monoclonal anti-Kir2.1 (1:100, ab109750, Abcam) primary antibodies were used. These antibodies bind to extracellular domains of Nav1.5 (residues 59-72 of human Nav1.5) and Kir2.1 (residues 107-122 of human Kir2.1 channels), respectively. Duolink secondary antibodies conjugated to PLA probes were also used. For electrophysiological experiments, rabbit anti-Nav1.5 (1.2 μg/mL, S0819, Sigma) and rabbit anti-Kir2.1 (1:1,000, ab65796, Abcam) were used. Both Nav1.5 (residues 493–511 located at the cytoplasmic loop between Domain I and II) and Kir2.1 (residues 1–81) antibodies bind to intracellular domains of the channels. For co-immunoprecipitation and western blot analysis rabbit polyclonal anti-CAMKIIδ (1:5,000 A010-56AP, Badrilla, UK) and rabbit polyclonal anti-GFP (1:1,000 ab290; Abcam) primary antibodies were used for detection of the C-terminal domain of CAMKIIδ and Kir2.1-CpVenus, respectively. Peroxidase-conjugated secondary antibodies (Jackson ImmunoResearch, USA) were used for detection of the proteins in lysates and HRP-protein A (Life Technologies) was used for detection of the immunoprecipitated proteins. Non-immune antibodies (IgG from rabbit or mouse serum) used as negative controls for co-immunoprecipitation experiments were obtained from Sigma-Aldrich.

### Statistical analysis

Results are expressed as mean ± SEM. Unpaired t-test or one-way ANOVA followed by Newman–Keuls test were used where appropriate. In small-size samples (*n* < 15), statistical significance was confirmed by using non-parametric tests. To take into account repeated sample assessments, data were analyzed with multilevel mixed-effects models. A value of *P* < 0.05 was considered significant.

## Results

**Figure 1:**
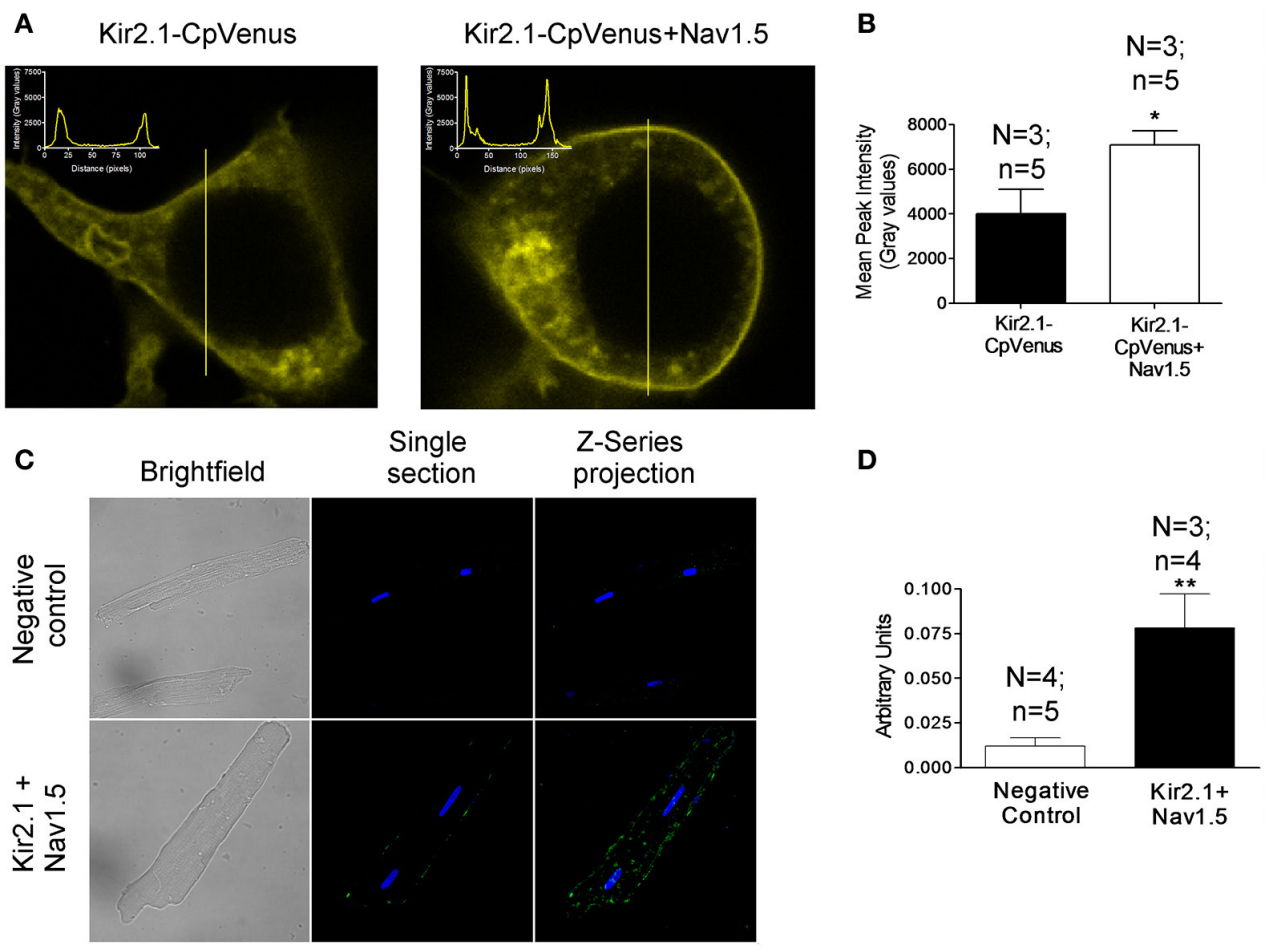
Kir2.1 and Nav1.5 channels are in close proximity at the membrane of cardiac myocytes. **(A)** Confocal microscopy images of HEK293 cells expressing Kir2.1-CpVenus alone (left) or in the presence of Nav1.5 (right). The insets show the intensity of the signal emitted by CpVenus throughout the cell region selected (yellow line). **(B)** Peak mean signal corresponding to the cell membrane in cells expressing Kir2.1-CpVenus with or without Nav1.5 channels. **(C)** Confocal microscopy images of rat ventricular myocytes processed using Duolink® PLA. The green signal demonstrates a physical interaction between Nav1.5 and Kir2.1. Cell nuclei were visible by DAPI staining (blue). **(D)** Mean intensity of the green signal in the negative control and in the presence of Nav1.5+Kir2.1 antibodies. In **(B,D)**, each bar represents the mean ± SEM of “*n*” cells of “*N*” preparations. ^*^P < 0.05 vs. Kir2.1-CpVenus alone. ^**^P < 0.01 vs. negative control.

The positive reciprocal modulation between Kir2.1 and Nav1.5 channels is not due to a change of the biophysical properties, such as single channel conductance or open probability of the channels (Milstein et al., 2012). These results led to propose that it is due to an increase of their expression at the cell membrane. To test this hypothesis, we conducted immunofluorescence analyses in HEK-293 cells transfected with Kir2.1 alone or together with Nav1.5 channels. Figure 1A left shows representative z-stack confocal images obtained in a cell expressing Kir2.1-CpVenus alone. The intensity of the signal emitted by CpVenus throughout the cell region selected (yellow line) has been represented as an inset (top left). The maximum signal, which corresponds to the cell membrane, is represented by the two discrete peaks. As can be observed in the right panel of Figure 1A and Figure 1B, coexpression with Nav1.5 channels significantly increased the intensity of the maximum signal emitted by CpVenus. Even though considering the limitations of the experimental approach, these results suggested that the amount of Kir2.1 channels at the cell membrane increases (*P* < 0.05).

Next, we measured the putative association between Nav1.5 and Kir2.1 channels using PLA in rat ventricular myocytes by means of the Duolink® technology. In PLA, a fluorescent signal is only generated when the plus and minus probes attached to each antibody are bound together, allowing for the *in situ* detection of proteins that are located in close proximity to each other (<40 nm apart; Söderberg et al., 2006; Shy et al., 2014). Importantly, to conduct these experiments myocytes were not permeabilized and, thus, the antibodies, which bind to extracellular domains of Nav1.5 and Kir2.1 channels (see section Methods), only detected the Nav1.5 and Kir2.1 channels present at the cell membrane. Figure 1C (bottom middle) shows a single section image from the center of a rat ventricular myocyte showing a sparse fluorescent signal distributed along the cell membrane. The Z-series projection (bottom right) shows more abundant fluorescent spots corresponding to the top of the membrane. Comparison of the mean intensity data demonstrated a ≈6.5-fold increase in the fluorescence signal (Figure 1D) compared to the negative controls (Figure 1C top panels) consisting of myocytes assayed with Duolink® technology but without incubating with the primary Nav1.5 and Kir2.1 antibodies (*P* < 0.01). Further negative controls were developed in human atrial myocytes in which PLA was developed after extracellular treatment with either anti-Kir2.1 or anti-Nav1.5 antibodies alone. Supplemental Figure 1 confirms that in the absence of one of the antibodies the Duolink assay did not produce any fluorescent signal. These data demonstrated that at the cardiomyocyte membrane at least a pool of Nav1.5 and Kir2.1 channels physically interacts with each other.

**Figure 2:**
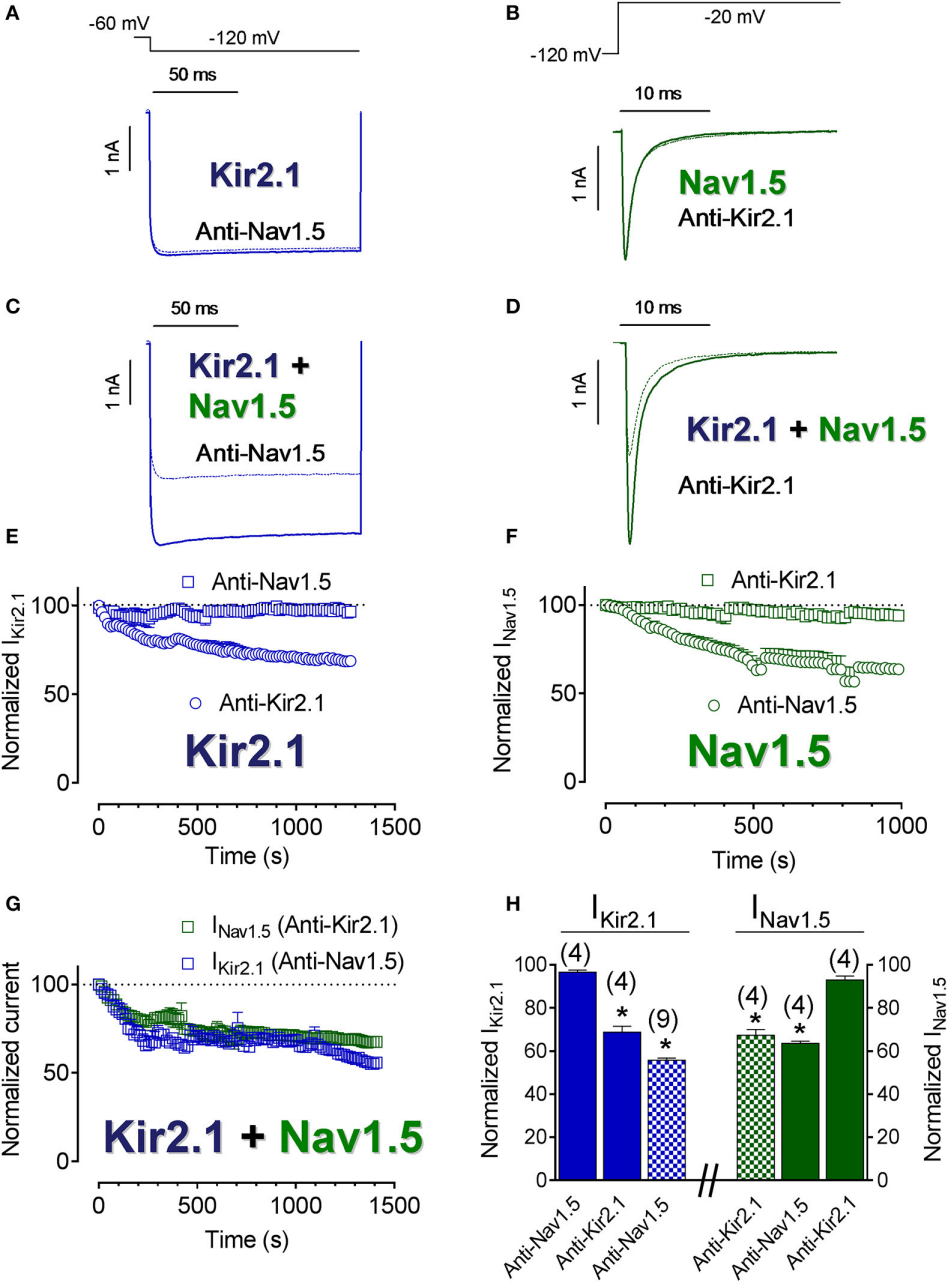
Kir2.1 and Nav1.5 channels closely interact at the cell membrane. **(A–D)** I_Kir2.1_ **(A,C)** and I_Nav1.5_ **(B,D)** traces recorded by applying the protocols shown at the top in CHO cells expressing Kir2.1 or Nav1.5 channels alone **(A,B)** or together **(C,D)** in control conditions (solid lines) or after dialyzing the cell with the antibodies indicated (dashed lines). **(E–G)** Normalized I_Kir2.1_ (measured at −120 mV) **(E,G)** and peak I_Nav1.5_ **(F,G)** measured in the absence and presence of anti-Kir2.1 or anti-Nav1.5 antibodies in cells transfected with Kir2.1 or Nav1.5 channels alone **(E,F)** or cotransfected with both **(G)** plotted as a function of time. **(H)** Bar graphs showing the mean normalized current density values measured in each experimental group at the end of the recordings. Each point/bar represents the mean ± SEM of (*n*) cells of ≥ 3 preparations. In **(H)**, ^*^P < 0.05 vs. I_Kir2.1_ or I_Nav1.5_ generated by Kir2.1 or Nav1.5 channels alone in the presence of anti-Nav1.5 or anti-Kir2.1 antibodies. For clarity the results of other statistical comparisons were not shown.

To further elucidate the degree of proximity of Kir2.1 and Nav1.5 channels at the cell membrane, the I_Kir2.1_ and I_Nav1.5_ were recorded in CHO cells transfected with Kir2.1, Nav1.5 channels or both and dialyzed with an internal solution supplemented with either anti-Kir2.1 or anti-Nav1.5 antibodies. In these experiments, the tip of the pipette was filled with antibody-free internal solution, in order to obtain “control” current records. Importantly, the epitope of the anti-Nav1.5 and anti-Kir2.1 antibodies used in these experiments (see section Methods) were intracellular domains of Nav1.5 and Kir2.1 channels, respectively. Figures 2E,F show the normalized density of I_Kir2.1_ recorded at −120 mV and of peak I_Nav1.5_ as a function of time elapsed after seal breaking and, thus of time in the presence of their corresponding antibodies. As expected, neither I_Kir2.1_ (Figures 2A,E) nor I_Nav1.5_ (Figures 2B,F) decreased in cells dialyzed with anti-Nav1.5 or anti-Kir2.1 antibodies (squares), respectively (Figure 2H). Conversely, and also as expected, both I_Kir2.1_ (Figure 2E) and I_Nav1.5_ (Figure 2F) progressively decreased in cells dialyzed with anti-Kir2.1 and anti-Nav1.5 antibodies (circles), respectively (*P* < 0.05; Figure 2H). Figures 2C,D show superimposed I_Kir2.1_ (Figure 2C) and I_Nav1.5_ (Figure 2D) traces recorded in cells cotransfected with Kir2.1+Nav1.5 channels and dialyzed with anti-Nav1.5 and anti-Kir2.1 antibodies, respectively, while Figure 2G shows the normalized density of I_Kir2.1_ and of peak I_Nav1.5_ as a function of dialyzing time. Surprisingly, I_Kir2.1_ density decreased in cells dialyzed with anti-Nav1.5 antibodies and I_Nav1.5_ also diminished in cells dialyzed with anti-Kir2.1 antibodies (Figures 2C,D,G,H; *P* < 0.05). These results would suggest that the distance between both channels is narrow enough to allow Kir2.1 channels to be “silenced” when the Nav1.5 antibody interacts with the Nav1.5 channels present in the Kir2.1-Nav1.5 complexes and *vice versa* and further supported the hypothesis that at least a pool of Kir2.1 and Nav1.5 channels interact at the cell membrane forming complexes.

### Effects of CaMKII on Kir2.1 and Nav1.5 channels and Kir2.1-Nav1.5 complexes

**Figure 3:**
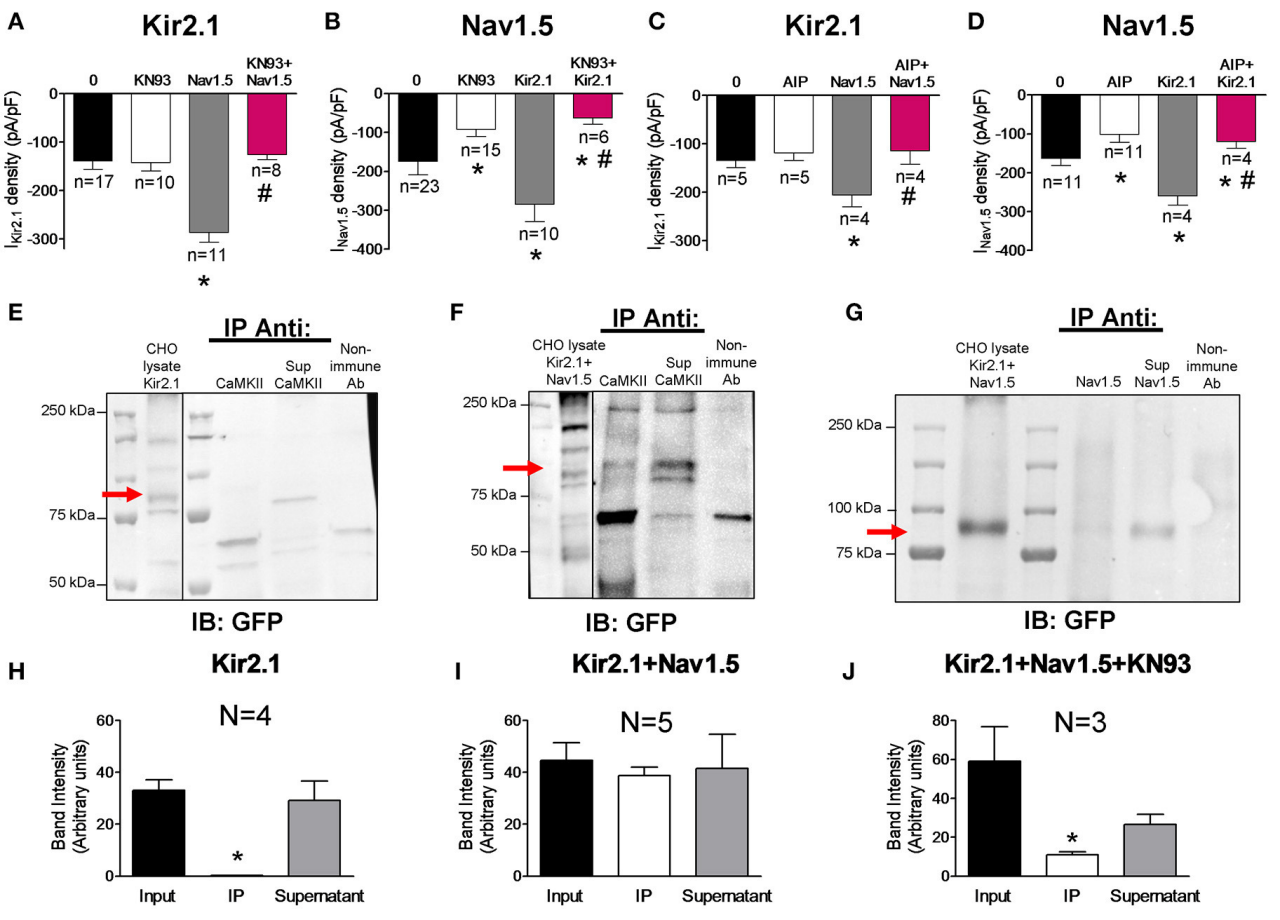
Effects of CaMKII inhibition on Kir2.1 and Nav1.5 channel complexes. **(A,B)** I_Kir2.1_ density at −120 mV **(A)** and peak I_Nav1.5_ **(B)** recorded in CHO cells expressing the constructs indicated, incubated or not with KN93 (1 μM-24 h). **(C,D)** I_Kir2.1_ density at−120 mV **(C)** and peak I_Nav1.5_ **(D)** recorded in CHO cells expressing the constructs indicated, dialyzed or not with AIP (1 nM). **(E–G)** Kir2.1 did not immunoprecipitate with CaMKII in the lysates obtained from CHO cells transfected with Kir2.1-CpVenus alone **(E)**, while partially immunoprecipitated in cells cotransfected with Nav1.5 channels **(F)**. In the presence of KN93 (1 μM-24 h incubation), Kir2.1 did not immunoprecipitate with Nav1.5 **(G)**. CpVenus was detected by western blot in the lysates by using an anti-GFP antibody. Also shown is the supernatant (Sup) recovered after centrifugation of the immunoprecipitant (IP). All immunoprecipitation reactions used membrane-enriched preparations. As a negative control, lysate treated with non-specific IgG (and Protein A/G) was used (“Non-immune Ab” lane). IB, antibody used for immunoblotting; IP Anti, antibody used for immunoprecipitation. **(H–J)** Densitometric analysis of the co-immunoprecipitation experiments depicted in **(E–G)** showing the mean intensity of the bands of the inputs, immunoprecipitants (IP) and supernatants. In **(A–D)** and **(H–J)**, each bar represents the mean ± SEM of “*n*” cells of ≥2 preparations **(A–D)** or “*N*” preparations **(H–J)**. In **(A–D)**, ^*^P < 0.05 vs. cells transfected with Kir2.1 or Nav1.5 channels alone; ^#^*P* < 0.05 vs. cells transfected with Kir2.1+Nav1.5. For clarity the results of other statistical comparisons were not shown. In **(H,J)**, ^*^P < 0.05 vs. input and supernatant.

It is well-established that Nav1.5 channels are one of the most important targets of CaMKII in cardiac myocytes (Wagner et al., 2006; Ashpole et al., 2012; Herren et al., 2015). Electrophysiological studies in HEK-293 cells have demonstrated that the kinase phosphorylates residues located in the cytoplasmic loop between domains I and II of the channel leading to an increase of the late I_Na_ (I_NaL_) and to a shift of the voltage-dependence of Nav1.5 channel inactivation to hyperpolarized potentials (Ashpole et al., 2012). To analyze the modulatory role of CaMKII on the channels expressed separately or together, we recorded the I_Kir2.1_ and I_Nav1.5_ in cells transfected with the channels and incubated or not with the CaMKII inhibitor KN93 (1 μM) for 24 h. Under our experimental conditions, inhibition of CaMKII did not modify I_Kir2.1_ in cells transfected with Kir2.1 alone (*n* ≥ 10; *P* > 0.05; Figure 3A) whereas it significantly decreased the I_Nav1.5_ in cells transfected with Nav1.5 channels alone (*P* < 0.05; Figure 3B). Furthermore, inhibition of the CaMKII-dependent phosphorylation significantly decreased the I_NaL_ recorded at the end of 500 ms pulses and shifted the inactivation curve to depolarized potentials (Supplemental Figure 2). Coexpression of Nav1.5 and Kir2.1 channels markedly and significantly increased both I_Kir2.1_ (Figure 3A) and I_Nav1.5_ (Figure 3B) (gray bars) as a consequence of the reciprocal positive modulation (Milstein et al., 2012; Matamoros et al., 2016), and the I_NaL_ (Supplemental Figure 2). Interestingly, in cells cotransfected with Kir2.1 and Nav1.5 channels simultaneously, incubation with KN93 significantly reduced the I_Nav1.5_ (Figure 3B) and I_NaL_ as expected (Supplemental Figure 2), and, unexpectedly, it also reduced the I_Kir2.1_ compared to that generated in cotransfected cells in which CaMKII was not inhibited (Figure 3A). In another group of experiments CaMKII was inhibited with autocamtide-2-related inhibitory peptide (AIP) which was added to the “internal” solution that dialyzes the cells after the patch rupture (1 nM). Data presented in Figures 3C,D and Supplemental Figure 2 confirmed that CaMKII inhibition significantly decreased the I_Nav1.5_ and the I_NaL_ in cells expressing Nav1.5 channels either alone or in the presence of Kir2.1 channels. Conversely, CaMKII inhibition only decreased the I_Kir2.1_ generated in cells cotransfected with Kir2.1 and Nav1.5 channels. Overall the results suggest that when both Kir2.1 and Nav1.5 channels are expressed together, a pool of Kir2.1 channels, probably those that are interacting with Nav1.5 channels, becomes a target for CaMKII (Figures 3B,D). To test this hypothesis co-immunoprecipitation experiments with Kir2.1-CpVenus channels cotransfected or not with Nav1.5 were conducted. As can be observed in Figures 3E,H, and consistently with the electrophysiological results, in cells expressing Kir2.1-CpVenus alone CaMKII did not co-immunoprecipitate with Kir2.1 channels, that were detected in the supernatant fraction by means of anti-GFP antibody which recognizes CpVenus. Conversely, CaMKII partially co-immunoprecipitated with Kir2.1 in cells cotransfected with both Kir2.1 and Nav1.5 channels (Figures 3F,I). The results pointed to a relevant role of CaMKII in the reciprocal positive modulation between Kir2.1 and Nav1.5 channels. Thus, we conducted co-immunoprecipitation experiments in cells cotransfected with Nav1.5 and Kir2.1 and incubated with KN93. Under these conditions Kir2.1-CpVenus channels did not co-immunoprecipitate with Nav1.5 channels (Figures 3G,J).

### Regulation of Kir2.1-Nav1.5 complexes by 14-3-3 proteins

**Figure 4:**
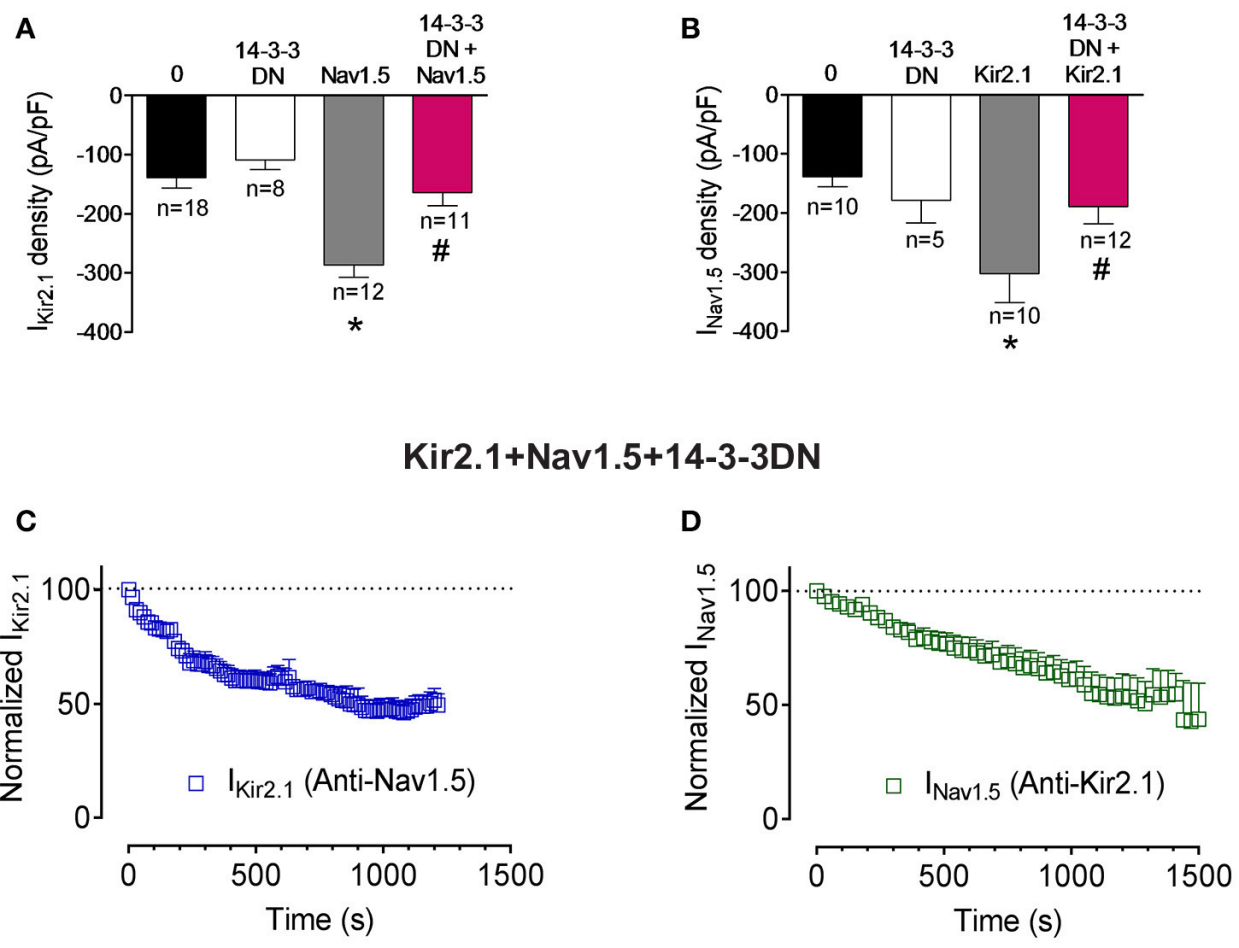
Effects of 14-3-3 inhibition on Kir2.1-Nav1.5 channel complexes. **(A,B)** I_Kir2.1_ density at −120 mV **(A)** and peak I_Nav1.5_ **(B)** recorded in CHO cells expressing the constructs indicated, cotransfected or not with 14-3-3DN. Each bar represents the mean±SEM of “*n*” cells of ≥ 4 preparations. ^*^P < 0.05 vs. cells transfected with Kir2.1 or Nav1.5 channels alone; ^#^*P* < 0.05 vs. cells transfected with Kir2.1+Nav1.5. For clarity the results of other statistical comparisons were not shown. **(C,D)** Normalized I_Kir2.1_ measured at −120 mV **(C)** and peak I_Nav1.5_ **(D)** measured in the absence and presence of anti-Nav1.5 and anti-Kir2.1 antibodies, respectively, in cells cotransfected with Kir2.1, Nav1.5, and 14-3-3DN plotted as a function of time. Each point represents the mean ± SEM of 4 experiments of ≥3 preparations in each group.

14-3-3 is a family of 30-kDa cytosolic acidic proteins with multiple effects including regulation of ion channel trafficking (Obsil and Obsilova, 2011). Furthermore, it has been demonstrated that 14-3-3 could prevent phosphatase action on phosphorylated residues (Tutor et al., 2006; Obsil and Obsilova, 2011). It has been shown that 14-3-3η can interact with Nav1.5 channels at the intercalated discs of cardiac myocytes. Moreover, 14-3-3 in COS7 cells modify the voltage dependence of inactivation and recovery from inactivation kinetics, but not the I_Nav1.5_ density (Allouis et al., 2006). To test whether 14-3-3 regulate somehow Kir2.1, Nav1.5, and Kir2.1-Nav1.5 complexes we used a double mutant (p.R56A,60A) of 14-3-3 which acts as a dominant negative (14-3-3DN; Allouis et al., 2006). Cotransfection of 14-3-3DN in cells expressing Kir2.1 or Nav1.5 alone did not significantly modify either I_Kir2.1_ or I_Nav1.5_ densities (Figures 4A,B; P>0.05). Interestingly, 14-3-3DN abolished the positive reciprocal modulation thus inhibiting both the I_Kir2.1_ and I_Nav1.5_ in cells cotransfected with both channels (Figures 4A,B; *P* < 0.05). These results would suggest that 14-3-3 proteins may target Kir2.1 and Nav1.5 channel complexes in a different way that each channel separately. However, these results could also be explained considering that 14-3-3 proteins are responsible for the positive reciprocal modulation. Therefore, we analyzed whether the inhibition of the 14-3-3 proteins precludes the interaction between Nav1.5 and Kir2.1 channels. To this end, we recorded the I_Kir2.1_ and I_Nav1.5_ in cells expressing both channels, cotransfected with 14-3-3DN, and dialyzed with an internal solution containing anti-Nav1.5 or anti-Kir2.1 antibodies, respectively. As can be observed in Figures 4C,D, when 14-3-3 proteins are inhibited, intracellular dialysis with anti-Nav1.5 and anti-Kir2.1 antibodies significantly decreased the I_Kir2.1_ and I_Nav1.5_, respectively. These results strongly suggested that inhibition of 14-3-3 proteins did not preclude the formation of Nav1.5-Kir2.1 complexes.

### Trafficking of Nav1.5 and Kir2.1 complexes

**Figure 5:**
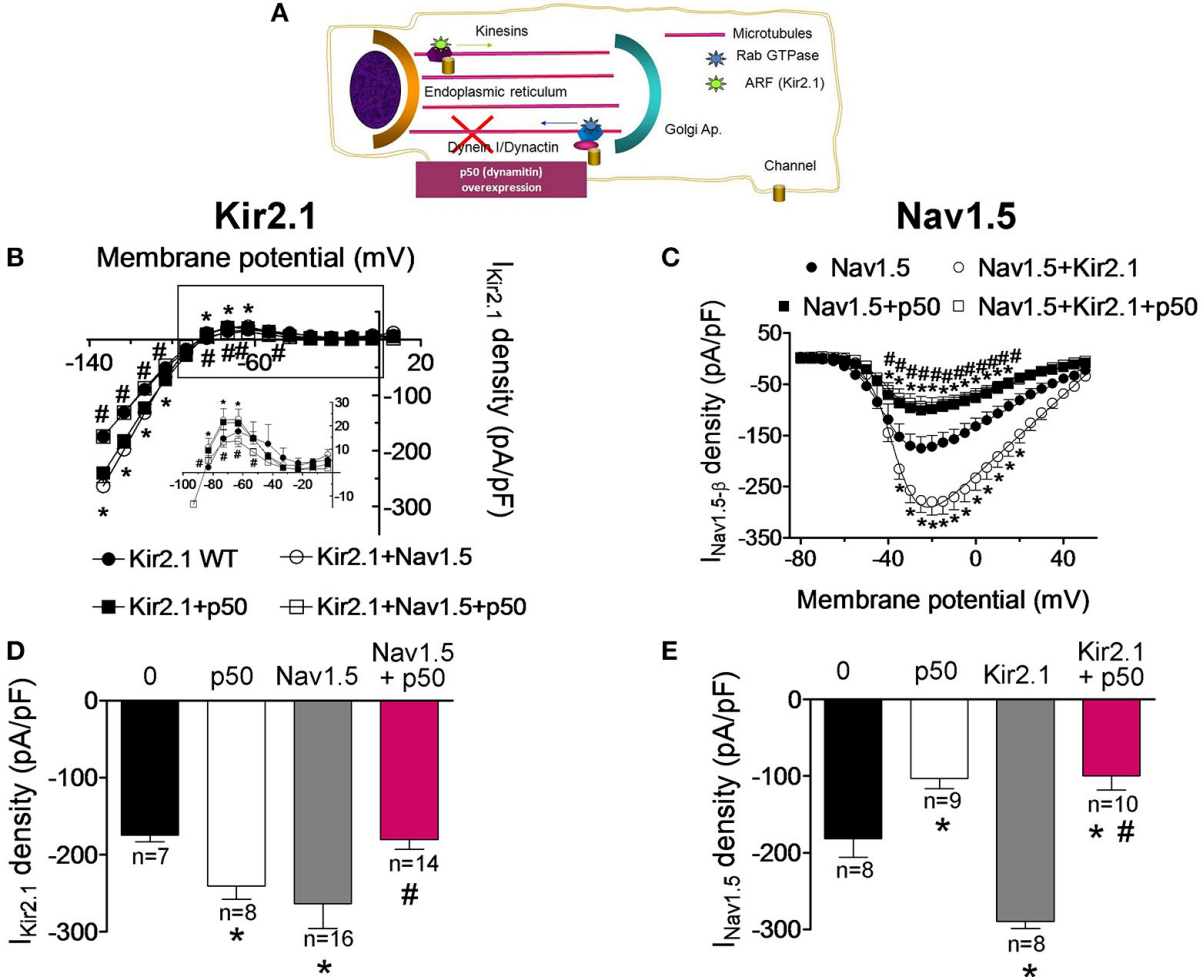
Effects of the dynein/dynactin complex inhibition on Kir2.1 and Nav1.5 channels trafficking. **(A)** Schematic representation of a cell highlighting the role of the dynein/dynactin complex on ion channel trafficking. **(B,C)** Current-density voltage relationships for I_Kir2.1_ **(B)** and I_Nav1.5_ **(C)** recorded in CHO cells expressing the constructs indicated together or not with dynamitin (the p50 subunit of dynactin) to inhibit the dynein/dynactin complex. **(D,E)** I_Kir2.1_ density at −120 mV **(D)** and peak I_Nav1.5_ **(E)** recorded in cells expressing the constructs indicated alone or together with p50. Each point/bar represents the mean ± SEM of “*n*” cells of ≥4 preparations. ^*^P < 0.05 vs. cells transfected with Kir2.1 or Nav1.5 channels alone; ^#^*P* < 0.05 vs. cells transfected with Kir2.1+Nav1.5 without p50. For clarity the results of other statistical comparisons were not shown.

In the next groups of experiments we further analyzed the biological properties of the Kir2.1-Nav1.5 complexes by comparing the trafficking of Kir2.1 and Nav1.5 channels when they are expressed alone or together in CHO cells. It has been proposed that the dynein/dynactin complex plays a role in normal vesicular traffic of ion channels toward and from the sarcolemma (Balse et al., 2012). Overexpression of dynamitin (the p50 subunit of dynactin) has been routinely used to block the dynein function since it dissociates the dynactin complex, decoupling the dynein motor from its cargo (Figure 5A; Burkhardt et al., 1997; Balse et al., 2012). Figures 5B,C show the current-density voltage curves for I_Kir2.1_ and I_Nav1.5_ recorded in cells cotransfected or not with p50. Cotransfection of p50 increased the I_Kir2.1_ at potentials between −120 and −90 mV and between −70 and −50 mV (Figures 5B,D; *P* < 0.05), suggesting that the dynein/dynactin complex is involved in the retrograde trafficking of Kir2.1 channels. Conversely, p50 reduced the I_Nav1.5_ at potentials ranging −40 and +30 mV in cells expressing Nav1.5 channels alone (Figures 5C,E; *P* < 0.05), thus confirming previous results that demonstrate the role of the dynein/dynactin complex in the anterograde trafficking of Nav1.5 channels (Chatin et al., 2014). When Kir2.1 and Nav1.5 channels were expressed together, both I_Kir2.1_ and I_Nav1.5_ were significantly increased as expected (Figures 5B–E). Conversely, overexpression of p50 abolished the reciprocal modulation between Kir2.1 and Nav1.5 (Figures 5B–E). In fact, in cells expressing Kir2.1+Nav1.5 channels in which the dynein function was blocked the I_Kir2.1_ was not increased (Figures 5B,D; *P* > 0.05). On the other hand, the density of the I_Nav1.5_ recorded in cells expressing Nav1.5, Kir2.1, and p50 was smaller than that recorded in cells expressing either Nav1.5 channels alone or Kir2.1+Nav1.5 (Figures 5C,E; *P* > 0.05). These results indicate that Kir2.1 channels are affected differently by the blockade of the dynein/dynactin complex when they are expressed alone or in combination with Nav1.5 channels and suggest that the dynein/dynactin complex is involved in the anterograde trafficking of Nav1.5 channels alone and of the Kir2.1-Nav1.5 channel complexes.

### Internalization and retrograde trafficking of Nav1.5 and Kir2.1 complexes

**Figure 6:**
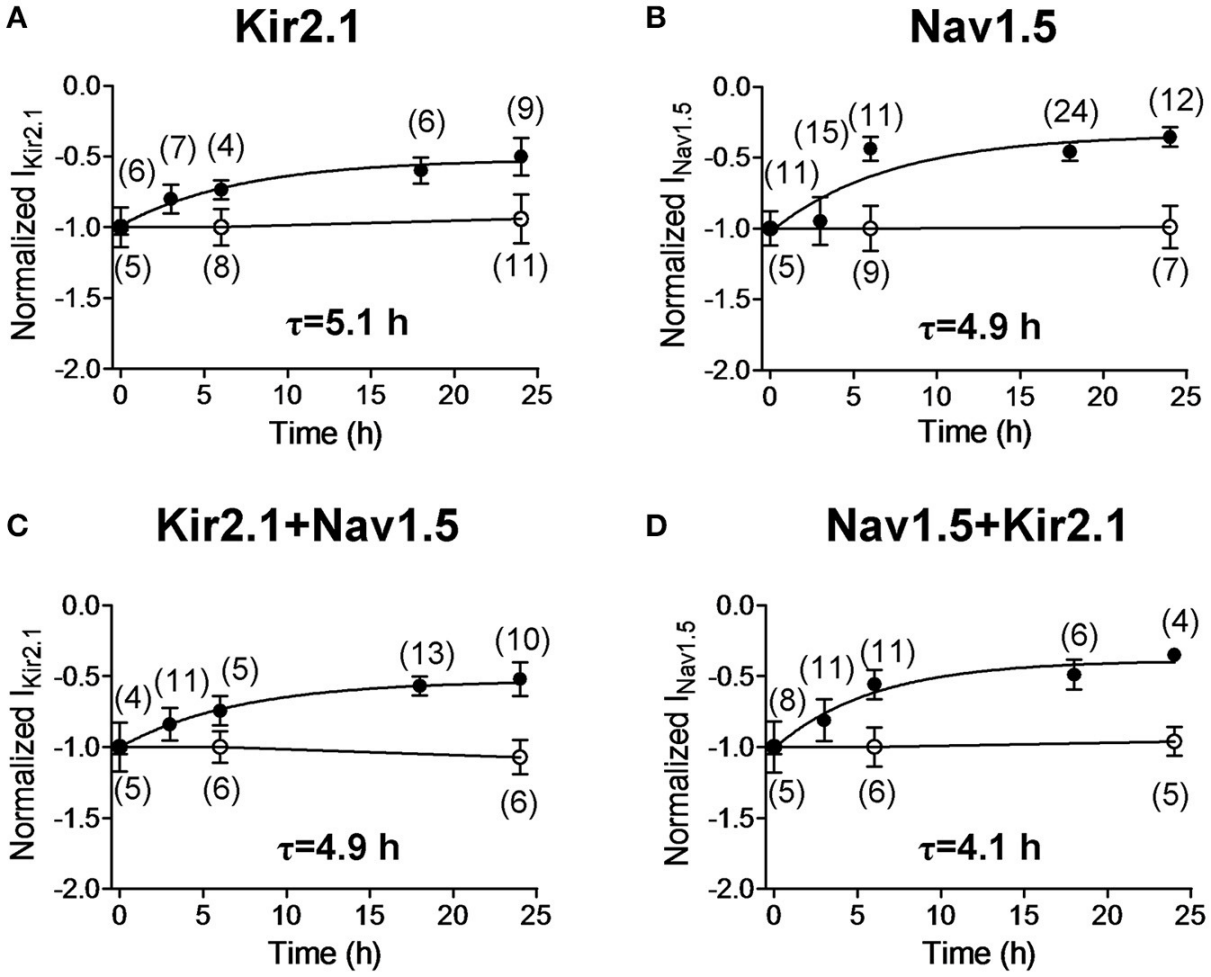
Time course of internalization of Kir2.1 and Nav1.5 channels. **(A–D)** Mean normalized density of the currents recorded in CHO cells expressing the constructs indicated and incubated with BFA (50 ng/mL) (solid circles) for 0 (Control), 3, 6, 18, and 24 h or with 1% ethanol (open circles) for 0, 6, and 24 h. Half-time constants (τ) yielded by the monoexponential fit to the BFA data are also shown. Each point represents the mean ± SEM of (*n*) cells of ≥4 preparations.

BFA is a fungal metabolite commonly used to block protein trafficking to cell membrane through the classical secretory pathway involving the endoplasmic reticulum (ER) and the Golgi apparatus (Rougier et al., 2005; Mercier et al., 2015). To test whether the Nav1.5 and Kir2.1 internalization kinetics are affected by the presence of both channels together, I_Nav1.5_ and I_Kir2.1_ were recorded after incubation with BFA (50 ng/mL) for progressively increasing periods of time. Using this approach, it would be expected that most of the trafficking of newly synthesized channels to the cell membrane is blocked, and thus, the time course of internalization of the channels located in the membrane can be assessed. Figures 6A–D show the normalized mean density of I_Kir2.1_ and I_Nav1.5_ recorded in CHO cells as a function of the duration of the incubation with BFA and, as can be observed, both I_Kir2.1_ and I_Nav1.5_ densities gradually decreased to a residual value upon incubation with BFA. In contrast, I_Kir2.1_ and I_Nav1.5_ densities did not change when cells were incubated with 1% ethanol, the BFA vehicle, for 6 or 24 h (Figures 6A–D). The fit of a monoexponential function to the data obtained in the presence of BFA yielded the half-time constants (τ) which for Kir2.1 and Nav1.5 channel internalization averaged 5.1 and 4.9 h, respectively (Figures 6A,B). In cells cotransfected with Kir2.1 and Nav1.5 channels the time course of Kir2.1 and Nav1.5 channel internalization was not significantly modified (Figures 6C,D; *n* ≥ 4; *P* > 0.05).

**Figure 7:**
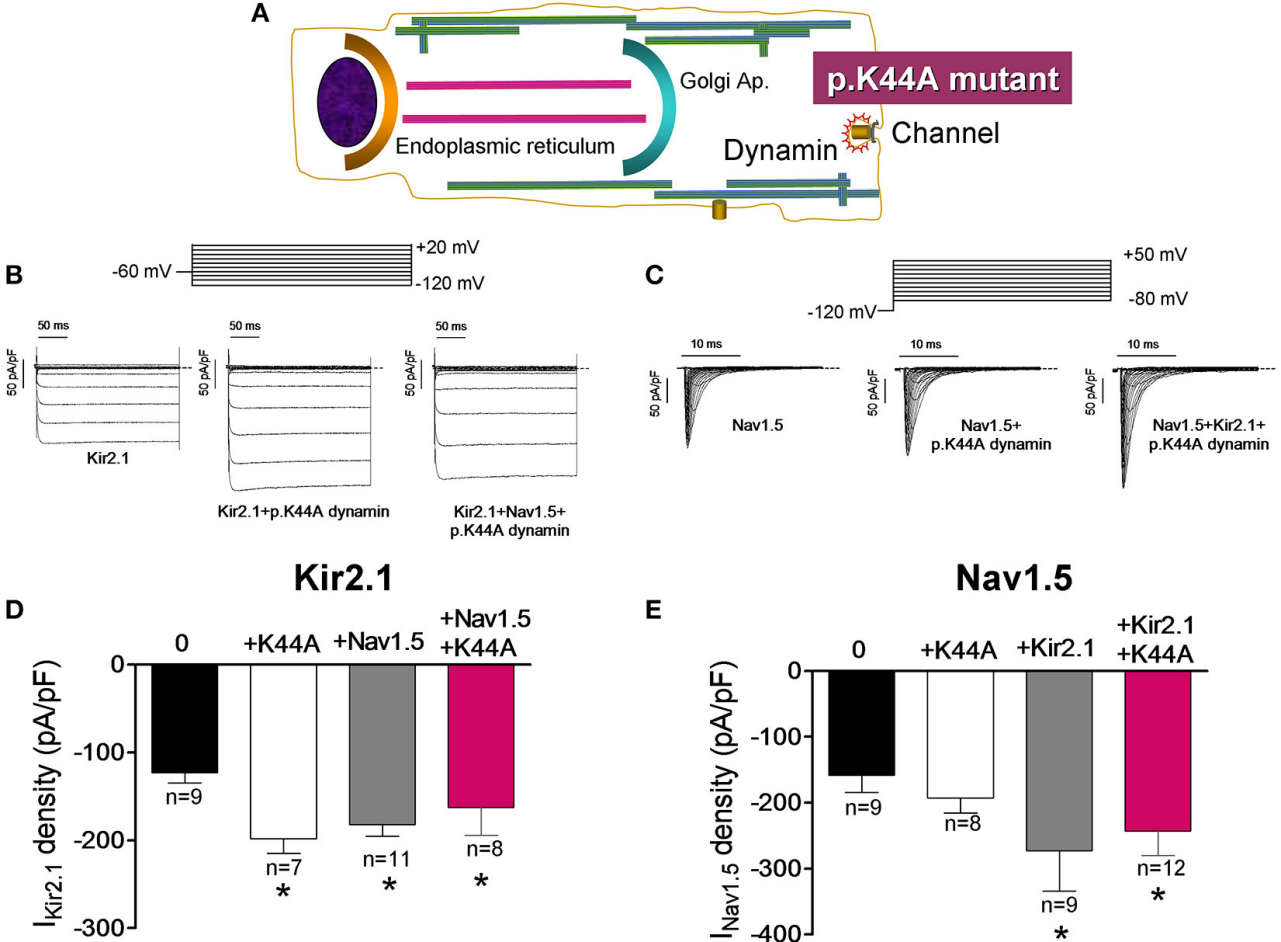
Effects of the dynamin-2 inhibition on Kir2.1 and Nav1.5 channels internalization. **(A)** Schematic representation of a cell highlighting the role of dynamin-2 in ion channel endocytosis. **(B,C)** I_Kir2.1_ **(B)** and I_Nav1.5_ **(C)** traces recorded by applying the protocols shown at the top in CHO cells transfected with the constructs indicated. The dashed lines represent the zero current level. **(D,E)** Bar graphs depicting I_Kir2.1_ density at −120 mV **(D)** and peak I_Nav1.5_ **(E)** in cells expressing Kir2.1 and Nav1.5 channels, respectively in the absence or presence of the constructs indicated cotransfected or not with p.K44A dynamin-2. Each bar represents the mean ± SEM of “n” experiments of ≥3 preparations. ^*^P < 0.01 vs. cells transfected with Kir2.1 or Nav1.5 channels alone. For clarity the results of other statistical comparisons were not shown.

It has been demonstrated that dynamin-2 has an important role in the endocytosis of different ion channels including Kir2.1 (Tong et al., 2001; Boyer et al., 2009). To test whether the Kir2.1 channel internalization is modified in the presence of Nav1.5 channels we took advantage of a dynamin-2 mutant (p.K44A; Figure 7A), which functions as a dominant negative and inhibits the activity of the endogenous dynamin-2 (Hinshaw and Schmid, 1995). In Figures 7B,C I_Kir2.1_ and I_Nav1.5_ traces recorded in CHO cells transfected with Kir2.1, Nav1.5 alone or together and cotransfected with p.K44A dynamin-2 are presented. In cells expressing Kir2.1 (Figures 7B,D) or Nav1.5 (Figures 7C,E) channels separately, overexpression of p.K44A dynamin-2 significantly increased the I_Kir2.1_ but did not modify the I_Nav1.5_ density, confirming that internalization of Kir2.1, but not that of Nav1.5, channels is dynamin-dependent. Again, coexpression of Nav1.5 and Kir2.1 channels reciprocally and positively modulated the I_Kir2.1_ and the I_Nav1.5_ densities (Figures 7D,E). Interestingly, in cells expressing Kir2.1 and Nav1.5 channels, p.K44A dynamin-2 expression did not further increase either the I_Kir2.1_ or the I_Nav1.5_ densities (*P* > 0.05), suggesting that, as Nav1.5 channels alone, the Kir2.1-Nav1.5 complexes are resistant to the dynamin-2 inhibition (Figures 7D,E).

**Figure 8:**
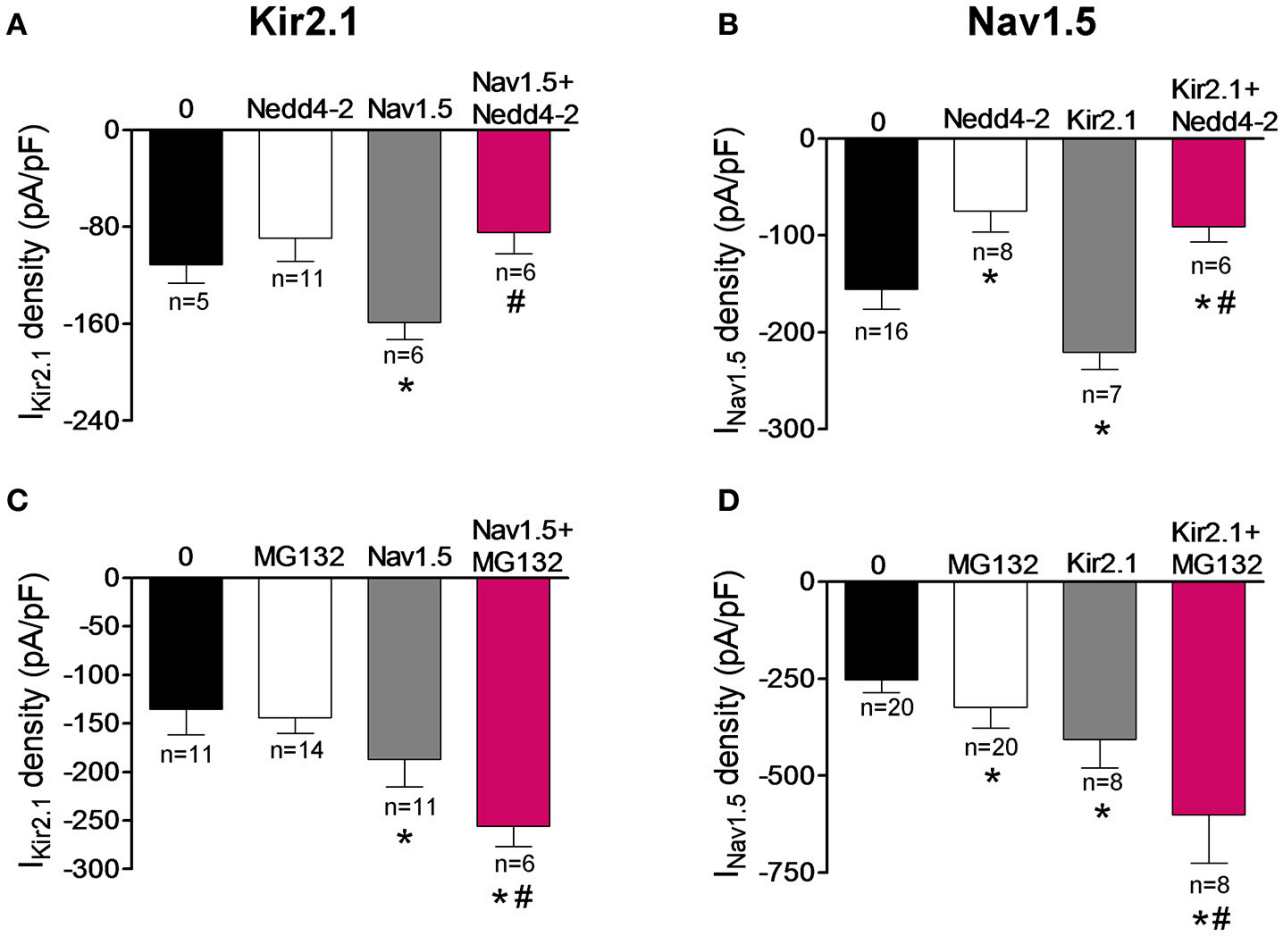
Kir2.1 and Nav1.5 channel complexes are ubyquitinated and degraded by the proteasome. **(A,B)** I_Kir2.1_ density at −120 mV **(A)** and peak I_Nav1.5_ **(B)** recorded in cells expressing Kir2.1 and Nav1.5 channels cotransfected or not with the ubiquitin ligase Nedd4-2. **(C,D)** I_Kir2.1_ density at −120 mV **(C)** and peak I_Nav1.5_ **(D)** recorded in cells expressing Kir2.1 and Nav1.5 channels incubated or not with the proteasome inhibitor MG132 (2 μM for 2 h). Each bar represents the mean ± SEM of “n” experiments of ≥4 preparations. ^*^P < 0.05 vs. cells transfected with Kir2.1 or Nav1.5 channels alone; ^#^*P* < 0.05 vs. cells transfected with Kir2.1+Nav1.5. For clarity the results of other statistical comparisons were not shown.

It has been extensively demonstrated that the ubiquitin-protein ligase Nedd4-2 ubiquitinates Nav1.5 by binding to the PY motif located at the C-terminus of the channel and promotes its degradation by the proteasome (van Bemmelen et al., 2004; Rougier et al., 2005; Xu et al., 2009). On the other hand, it was demonstrated that Nedd4-2 reduces the I_Kir2.1_ density in *Xenopus* oocytes, although the molecular determinants were not identified (Alesutan et al., 2011). The degradation route of the putative Kir2.1-Nav1.5 complexes was further analyzed by cotransfecting the cells with Nedd4-2. Under our conditions, Nedd4-2 did not significantly modify the I_Kir2.1_ in cells expressing Kir2.1 alone (Figure 8A; *P* > 0.05) but, as expected (van Bemmelen et al., 2004), it significantly decreased the I_Nav1.5_ in cells expressing Nav1.5 channels alone (*P* < 0.05; Figure 8B), confirming that Nav1.5, but not Kir2.1 channels, are targeted by Nedd4-2. Importantly, both I_Kir2.1_ and I_Nav1.5_ densities recorded in cells coexpressing Kir2.1, Nav1.5, and Nedd4-2 were significantly reduced (*P* < 0.05). These results indicated that Kir2.1-Nav1.5 complexes were targeted by Nedd4-2 as Nav1.5 channels were and suggested that these complexes are degraded by the proteasome. To test this hypothesis, I_Kir2.1_ (Figure 8C) and I_Nav1.5_ (Figure 8D) were recorded in cells incubated with the proteasome inhibitor MG132 (20 μM) for 2 h (Shy et al., 2014). MG132 treatment increased the I_Nav1.5_ in cells expressing Nav1.5 channels alone (Figure 8D; *P* < 0.05) without modifying the I_Kir2.1_ in cells expressing Kir2.1 channels alone (Figure 8C; *P* > 0.05). It could be possible that the absence of effects of MG132 on I_Kir2.1_ is due to the fact that degradation of Kir2.1 channels by the proteasome takes more time than that of Nav1.5 channels. To analyze this possibility, I_Kir2.1_ was recorded in cells incubated with MG132 for a longer period of time (4 h). Under these conditions, I_Kir2.1_ density was not significantly modified either (−159 ± 14 vs. −155 ± 18 pA/pF at −120 mV; *P* > 0.05). All these results are in agreement with the selective role of the proteosome in the degradation of Nav1.5 channels but not of Kir2.1 channels. In cells coexpressing Kir2.1 and Nav1.5 channels, incubation with MG132 produced a further increase of I_Kir2.1_ and I_Nav1.5_ densities compared to those generated by the positive reciprocal modulation (Figures 8C,D; *n* ≥ 6; *P* < 0.05). These results would confirm that Nav1.5 channels and Kir2.1-Nav1.5 complexes are degraded by the proteasome.

## Discussion

The present results suggest that there is a pool of Nav1.5 and Kir2.1 channels that form complexes that exhibit biological properties similar to those of Nav1.5 channels.

Previous data have shown that in ventricular myocytes there is a positive reciprocal modulation between Kir2.1 and Nav1.5 channels (Milstein et al., 2012; Matamoros et al., 2016) which is due to their interaction with α1-syntrophin (Matamoros et al., 2016). The results here obtained with immunofluorescence experiments in heterologous expression systems suggested that cotransfection with Nav1.5 channels markedly increased the expression of Kir2.1. Therefore, these results seem to add further support to the hypothesis that the Kir2.1-Nav1.5 reciprocal modulation relies on the increase in the expression of the channels in the cell membrane. On the other hand, the PLA here conducted demonstrated that Kir2.1 and Nav1.5 channels are in close proximity to each other at the membrane of cardiac cells (<40 nm). Moreover, when both channels are expressed together, intracellular targeting Kir2.1 with a specific antibody reduces the I_Nav1.5_ and *vice versa*. Therefore, we propose that there is a pool of Kir2.1 and Nav1.5 channels that form complexes (Milstein et al., 2012; Matamoros et al., 2016). Similarly, it has been shown that two voltage-dependent K^+^ channels, Kv11.1 (hERG) and Kv7.1 (KvLQT1), may interact in such a way that expression of Kv7.1 increases Kv11.1 currents without major biophysical changes (Ehrlich et al., 2004). Recently it has been shown that this modulation is not due to the formation of heterotetrameric Kv11.1-Kv7.1 channels, suggesting that the positive modulation was due to the interaction between both subunits (Biliczki et al., 2015) in a similar way as we propose for Nav1.5 and Kir2.1.

It has been demonstrated that CaMKII co-immunoprecipitates with and phosphorylates Nav1.5 (Wagner et al., 2006; Herren et al., 2015). This modulation acutely affects the channel gating increasing the I_NaL_ without a noticeable effect over the peak current in HEK-293 cells (Ashpole et al., 2012). In our hands inhibition of CaMKII decreased the I_NaL_ and shifted the voltage dependence of I_Nav1.5_ inactivation toward more positive potentials, results that are in agreement with those observed previously (Wagner et al., 2006). Additionally, here we demonstrated that long lasting CaMKII inhibition with KN93 or intracellular dialysis with AIP (two CaMKII inhibitors) decreased somehow the peak I_Nav1.5_. On the other hand, Wagner et al. (2009) demonstrated that CaMKII can produce dual effects on Kir2.1 channels, downregulating the mRNA and protein expression, but acutely increasing the I_K1_ density in rabbit and mouse myocytes. Our co-immunoprecipitation experiments showed that Kir2.1 channels alone did not co-immunoprecipitate with CaMKII, conversely, in cells expressing both Kir2.1+Nav1.5 channels they did. In agreement with these results, KN93 significantly reduced the I_Kir2.1_ when Kir2.1 and Nav1.5 channels are expressed together. Of note, in cardiac myocytes Nav1.5 and Kir2.1 channels are always expressed simultaneously, thus our results in transfection systems are in agreement with those obtained in native cells, i.e., CaMKII acutely increases I_Kir2.1_ and its inhibition diminishes the current density. It can be speculated that the presence of Nav1.5 together with Kir2.1 promotes a conformation of Kir2.1 channels that allows their interaction with the kinase (Wagner et al., 2006, 2009). Alternatively, it is possible that CaMKII-induced phosphorylation of Nav1.5 affects Kir2.1 when both channels form complexes. Furthermore, the coexpression with Kir2.1 channels significantly increased the I_NaL_ which, in turn, was diminished by the inhibition of CamKII. Moreover, when the CaMKII is inhibited, Kir2.1 channels did not co-immunoprecipitate with Nav1.5 channels anymore. Therefore, we propose that formation of Nav1.5-Kir2.1 complexes strengthens the interaction of CaMKII with both Kir2.1 and Nav1.5 channels, and, importantly, that CaMKII plays a role in the positive reciprocal modulation between Nav1.5 and Kir2.1 channels that merits to be elucidated.

**Figure 9:**
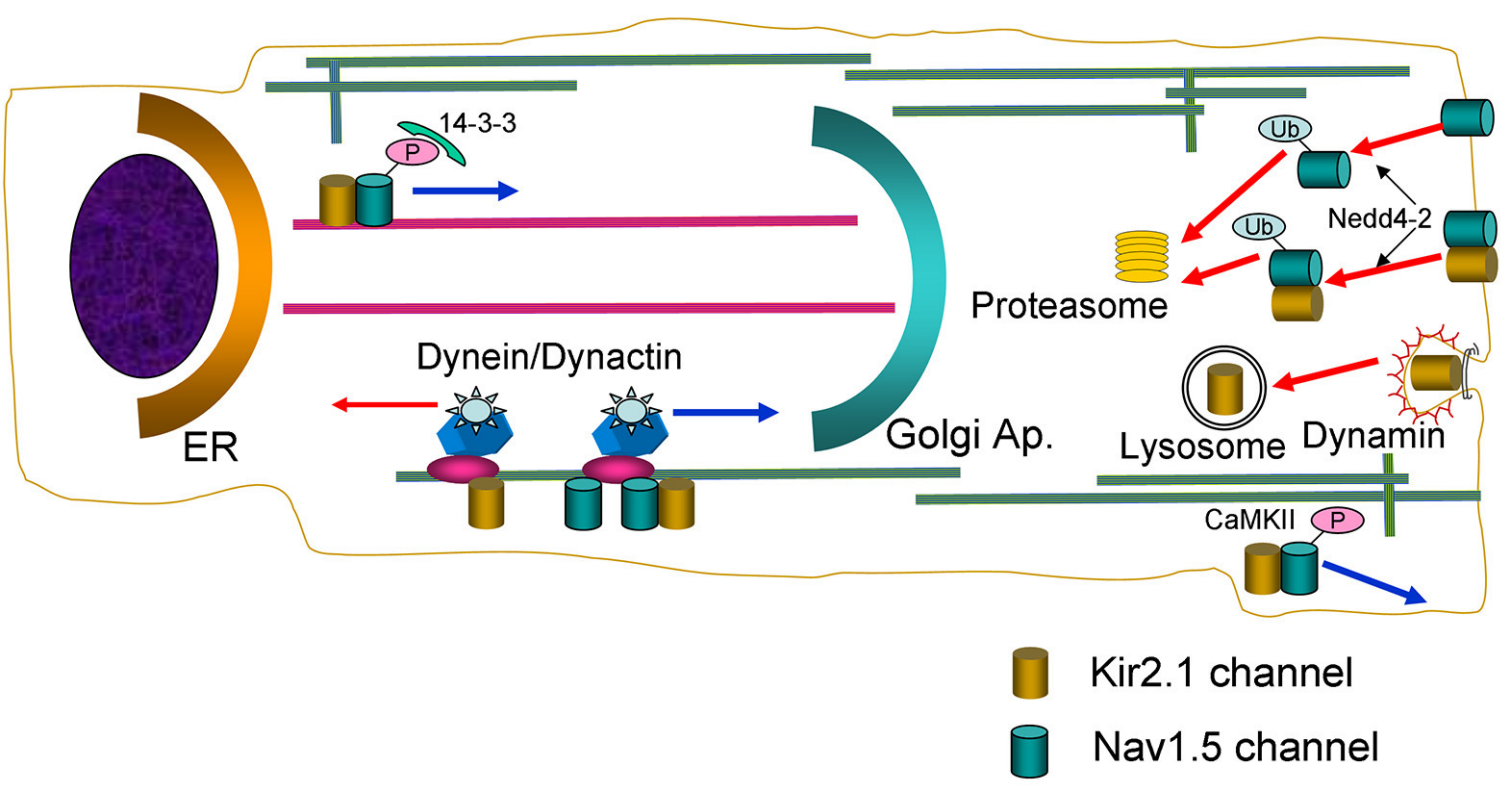
Schematic diagram summarizing the main findings of the present study. Nav1.5 and Kir2.1 channels are represented by green and golden cylinders, respectively. ER, endoplasmic reticulum; P, phosphate; Ub, ubiquitin chains.

It has been proposed that binding of 14-3-3 to ER retention motifs (RXR) decreases channel affinity for coat-associated protein I (COPI), an essential component for the recycling of membrane proteins from the Golgi to the ER, promoting surface expression of properly folded channels (Balse et al., 2012). In our hands, 14-3-3DN did not modify I_Kir2.1_ and I_Nav1.5_ densities in cells expressing the individual channels but inhibited both currents in cells expressing Kir2.1+Nav1.5 channels. These results would mean that the presence of both channels together promotes the existence of a structural conformation that allows the ER retention motifs (RXR) of the channel proteins to be accessible for 14-3-3 preventing their binding to COPI (Figure 9). It is also possible that the effects observed upon 14-3-3 inhibition could be the consequence of the disruption of the interaction between Kir2.1 and Nav1.5 channels, avoiding the formation of the complexes. However, the results obtained with the intracellular application of anti-Kir2.1 and anti-Nav1.5 antibodies suggested that 14-3-3 inhibition did not preclude the formation of Nav1.5-Kir2.1 complexes. It has been demonstrated that protein kinase A (PKA)-dependent phosphorylation of Nav1.5 channels enhances Nav1.5-trafficking from the ER toward the membrane in rat cardiomyocytes (Zhou et al., 2002). This effect seems to be mediated by the PKA phosphorylation of sites (S525 and S528) that are proximally-located to an ER retention signal (Zhou et al., 2002). On the other hand, 14-3-3 proteins preferentially bind to phosphorylated proteins and protect them from dephosphorylation, thus enhancing the effects derived from phosphorylation (Kagan et al., 2002; Tutor et al., 2006). Allouis and co-workers demonstrated that 14-3-3 proteins bind to the intracellular I to II linker of Nav1.5 channels, i.e., close to the PKA phosphorylation site (Allouis et al., 2006) and, thus, also probably protect Nav1.5 channels from dephosphorylation. Should this occur, binding of 14-3-3 proteins to Nav1.5 channels would increase the I_Na_ by enhancing the Nav1.5 trafficking from the ER toward the plasma membrane. Conversely, 14-3-3 binding to Nav1.5 channels does not increase the I_Na_ density (Allouis et al., 2006) as demonstrated in experiments conducted in hetereologous expression systems in which only Nav1.5 channels were transfected (Allouis et al., 2006 and the present results). It can be hypothesized that 14-3-3 proteins enhance the anterograde traffic of Nav1.5-Kir2.1 channel complexes, but not that of Nav1.5 channels alone. This would explain why inhibition of 14-3-3 proteins decreased the I_Nav1.5_ density in cells cotransfected with Nav1.5+Kir2.1 channels but not in cells transfected with Nav1.5 channels alone. Similarly, this would explain the I_Kir2.1_ increase observed in cells cotransfected with Nav1.5 and Kir2.1 channels. Further experiments are needed to test this hypothesis.

Overall, data here presented support the contention that at least a pool of Nav1.5 and Kir2.1 channels form complexes whose behavior is not identical to each channel separately (Figure 9). It has been described that in the brain Kir2.1 can form complexes with several anchoring and regulatory proteins such as SAP97 and Veli (Leonoudakis et al., 2004). However, the existence of such complexes in the heart has not been described thus far. Therefore, to further characterize the biology of the complexes, we analyzed how some of the mechanisms involved in anterograde and retrograde trafficking of the individual channels are affected when they are expressed together. Microtubule cytoskeleton is involved in ion channel trafficking by means of the dynein and kinesin motors that track along microtubules (Balse et al., 2012). The experiments conducted in cells overexpressing the p50 subunit to inhibit the dynein/dynamitin motor complex showed that it is involved in the retrograde and anterograde trafficking of Kir2.1 and Nav1.5 channels, respectively. Our results confirm previous data showing that p50 interacts with Nav1.5 channels reducing their expression at the cell surface and the I_Nav1.5_ density (Chatin et al., 2014). Importantly, our results also suggested that the dynein/dynamitin motor complex is critical for the anterograde trafficking of the pool of Kir2.1 and Nav1.5 channels that form the complexes, supporting the hypothesis that their biology may be similar to that of Nav1.5 channels (Figure 9).

The experiments conducted in cells incubated with BFA, demonstrated that internalization kinetics of Kir2.1-Nav1.5 complexes is similar to that of Kir2.1 and Nav1.5 channels alone. However, our results suggest that the internalization process of Kir2.1 channels is qualitatively different to that of the Kir2.1-Nav1.5 complexes. We demonstrated that dynamin-2 inhibition by overexpression of the p.K44A mutant increased the I_Kir2.1_, but not the I_Nav1.5_, confirming that Kir2.1 channel endocytosis is dynamin-dependent (Tong et al., 2001; Boyer et al., 2009), whereas that of Nav1.5 is not (Laedermann et al., 2014). Interestingly, p.K44A did not modify I_Kir2.1_ and I_Nav1.5_ generated in cells expressing both channels, suggesting that the internalization of Kir2.1-Nav1.5 complexes is not dynamin-dependent either (Figure 9). It has been shown that Nav1.5 channels internalization relies on ubiquitination by Nedd4-2 (van Bemmelen et al., 2004; Rougier et al., 2005; Laedermann et al., 2014). Data on Kir2.1 ubiquitination are scarce, although it was proposed that Nedd4-2 may reduce I_Kir2.1_ density in *Xenopus* oocytes (Alesutan et al., 2011). Our results confirmed that overexpression of Nedd4-2 reduces the I_Nav1.5_ but not the I_Kir2.1_ in cells expressing Nav1.5 and Kir2.1 channels alone, respectively. Interestingly, both I_Kir2.1_ and I_Nav1.5_ were reduced in cells expressing both Kir2.1 and Nav1.5 channels suggesting that the putative Kir2.1-Nav1.5 complexes are ubiquitinated by Nedd4-2 (Figure 9). It has been proposed that the fate of the ubiquitinated channels is different depending on whether they are mono- or polyubiquitinated. Monoubiquitinated proteins are more prone to enter a recycling pathway, whereas polyubiquitinated ones are preferentially degraded by the proteasome (Laedermann et al., 2014). Rougier et al. (2005) demonstrated that in HEK293 cells Nav1.5 channels are polyubiquitinated suggesting that they are subject to proteasomal degradation. Other studies, however, suggested that *in vivo* Nav1.5 could be mono- or polyubiquitinated (Laedermann et al., 2014). Our results suggested that Nav1.5 and Kir2.1-Nav1.5 complexes are polyubiquitinated and degraded by the proteasome (Figure 9).

### Physiological relevance

I_K1_ and I_Na_ play a key role in regulating cardiac excitability under physiological conditions and in the control of the frequency of the rotors that are responsible for fibrillating arrhythmias (Jalife, 2016). The classical paradigm considered that these currents were functionally coupled. Our results provide new evidences demonstrating that there is also a physical interaction between a pool of Nav1.5 and Kir2.1 channels that allows the formation of complexes, which probably are formed at early stages of protein assembly. By means of such a mechanism, the membrane expression of these ion channels in the ventricles can be strictly and simultaneously regulated, allowing a tighter control of the resting membrane potential and excitability and thus, preventing the ocurrence of fibrillating arrhythmias. Therefore, it can be speculated that changes in the expression of one of the channels, such as those produced by disease-associated remodeling or mutations, could concomitantly alter the expression of the other channel. We recently demonstrated that in human atrial myocytes obtained from patients in sinus rhythm and chronic atrial fibrillation (CAF) the CAF-induced I_K1_ increase was not accompanied by an I_Na_ increase. This result was explained considering that the presence of Kir2.3 subunits precludes the reciprocal positive modulation between Nav1.5 and Kir2.x channels (Matamoros et al., 2016) and that in the human atria Kir2.3 channels are preferentially expressed (Gaborit et al., 2007). Further studies are needed to analyze whether changes in the expression of Kir2.1 or Nav1.5 produced by disease-associated remodeling process (for instance the Kir2.1 decrease produced by heart failure) might be accompanied by reciprocal decreases of either the I_Na_ or the I_K1_ in the human ventricle. On the other hand, the consequences of the presence of mutated Nav1.5 or Kir2.1 channels on the ventricular I_K1_ and the I_Na_, respectively, must also be elucidated.

### Limitations of the study

Most of the results were obtained in heterologous expression systems and not in cardiac myocytes. This allowed us to compare separately the effects of each experimental maneuver on Nav1.5 and Kir2.1 channels as well as on the Kir2.1-Nav1.5 complexes. It is important to note that the vast majority of the information on cardiac ion channel biology has been obtained in expression systems (Balse et al., 2012). This could be accounted for considering the limited survival of cultured cardiac myocytes, since for the analysis of the channel biology the transfection with the cDNA encoding several proteins or the incubation with some reagents with high toxicity is needed. Our results stress the fact that results obtained in heterologous expression systems with isolated channels could be different to what actually occurs in cardiac myocytes where the rest of the channels are also present.

## Conclusion

Overall our results suggest that there is a pool of Nav1.5 and Kir2.1 channels that form complexes with anterograde and retrograde trafficking routes similar to those of Nav1.5 channels alone. These complexes allow a tight control of the current densities that determine cardiac excitability and action potential propagation. Therefore, the formation of the complexes would have an important physiological and/or pathological relevance that merits further investigation.

## Author contributions

Conducting experiments, acquiring and analyzing data, RGU, PN-M, SA, DT, MM, MP-H, SS, LO, RdA, and FJD-G. Designing research, analyzing data, and writing MS: JT, ED, and RC.

### Conflict of interest statement

The authors declare that the research was conducted in the absence of any commercial or financial relationships that could be construed as a potential conflict of interest. The reviewer JR and handling Editor declared their shared affiliation. The handling editor declared a past collaboration with the authors MM and MP-H.

## Abbreviations

AIP: autocamtide-2-related inhibitory peptide
BFA: Brefeldin-A
CAF: chronic atrial fibrillation
CaMKII: Ca^2+^/calmodulin-dependent protein kinase II
CHO: Chinese Hamster Ovary
COPI: coat-associated protein I
DAPI: 4',6-diamidino-2-phenylindole
ER: Endoplasmic reticulum
HEK-293: Human Embryonic Kidney-293
I_K1_: Inward rectifier potassium current
I_Kir2.1_: Current generated by Kir2.1 channels
I_Na_: Inward sodium current
I_NaL_: late sodium current
I_Nav1.5_: Current generated by Nav1.5 channels
I-V: current-voltage
LJP: Liquid junction potential
PBS: Phosphate buffered saline
PKA: protein kinase A
PLA: proximity ligand assay
TRITC: Tetramethylrhodamine.
