# Supplementary material for: Kir2.1-Nav1.5 Channel Complexes Are Differently Regulated than Kir2.1 and Nav1.5 Channels Alone
Source: Front Physiol. 2017 Nov 14;8:903. doi: 10.3389/fphys.2017.00903 (PMC5694551; doi:10.3389/fphys.2017.00903)
Supplement: Supplementary file 1 [file Image1.PDF]

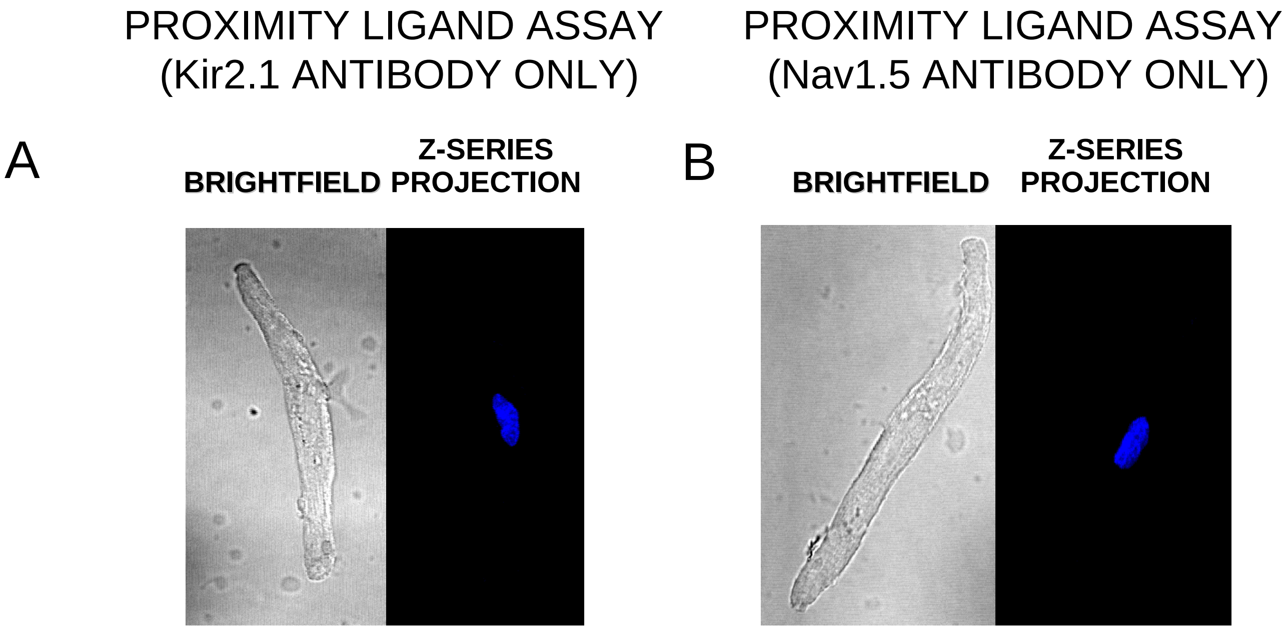

Supplemental Figure 1. A and B. Confocal microscopy images of human atrial myocytes processed using Duolink® PLA when omitting Nav1.5 (A) or Kir2.1 (B) primary antibodies. Cell nuclei were visible by DAPI staining (blue). As can be observed, when only Kir2.1 (A) or Nav1.5 (B) primary antibodies were used no PLA signal could be detected, ruling out the existence of non-specific interactions.
