# Supplementary material for: Kir2.1-Nav1.5 Channel Complexes Are Differently Regulated than Kir2.1 and Nav1.5 Channels Alone
Source: Front Physiol. 2017 Nov 14;8:903. doi: 10.3389/fphys.2017.00903 (PMC5694551; doi:10.3389/fphys.2017.00903)
Supplement: Supplementary file 2 [file Image2.PDF]

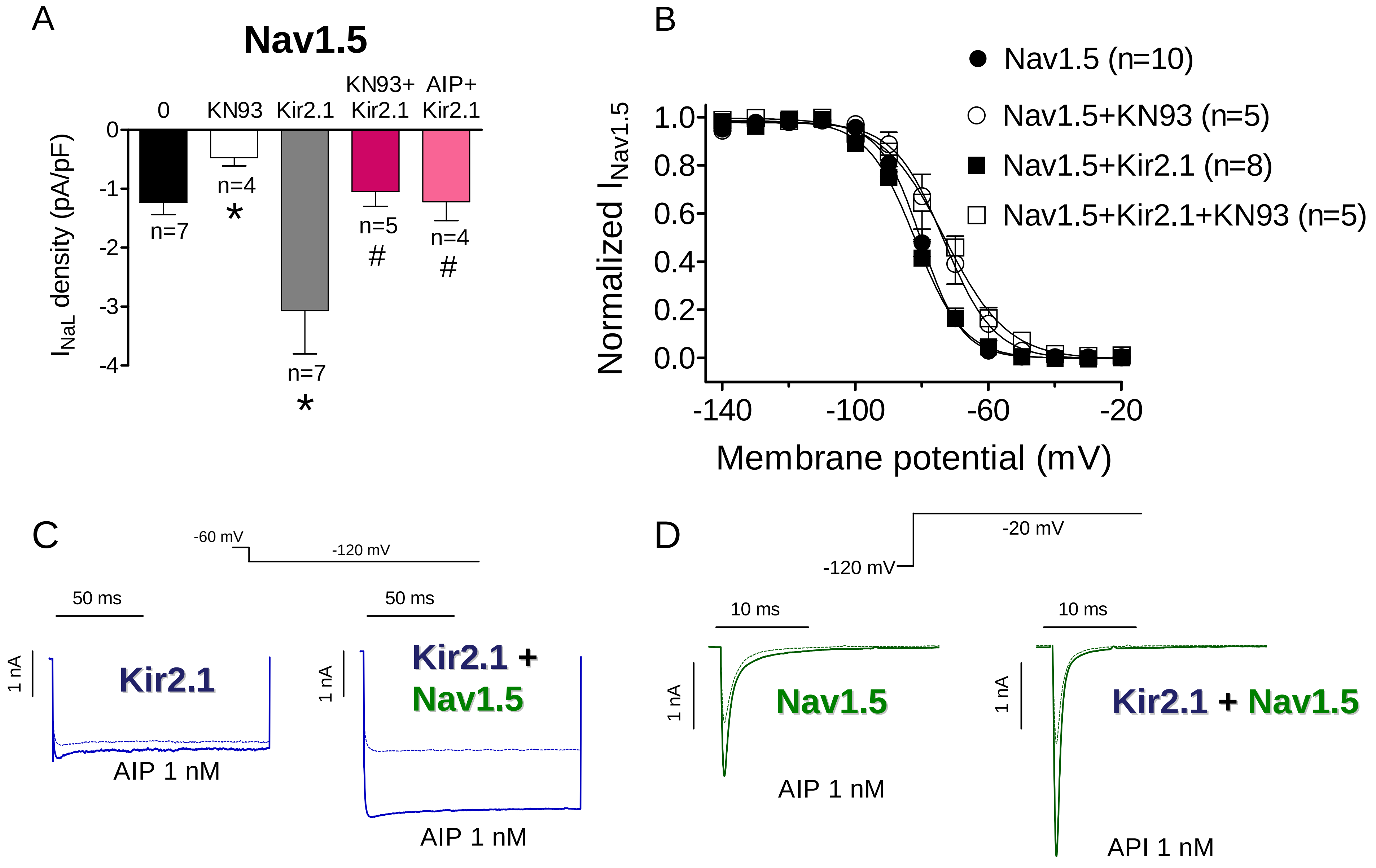

Supplemental Figure 2

Supplemental Figure 2. A. Mean density of the I_{NaL} recorded in CHO cells expressing the constructs indicated, incubated or not with KN93 (1 μM-24 h) or dialyzed with AIP (1 nM). **B.** Steady-state inactivation curves of I_{Na} recorded in CHO cells expressing the constructs indicated, incubated or not with KN93 (1 μM-24 h). The solid lines represent the fit of a Boltzmann function to the data points. **C and D.** $I_{Kir2.1}$ (C) and $I_{Nav1.5}$ (D) traces recorded by applying the protocols shown at the top in CHO cells expressing Kir2.1 or Nav1.5 channels alone (left panels) or together (right panels) in control conditions (solid lines) or after dialyzing the cell with 1 nM AIP (dashed lines). In A and B, each bar/point represent the mean±SEM of (n) cells. In A, *P<0.05 vs cells transfected with Kir2.1 or Nav1.5 channels alone; #P<0.05 vs cells transfected with Kir2.1+Nav1.5. For clarity the results of other statistical comparisons were not shown.

As can be observed in Panel A, KN93 or AIP significantly decreased the I_{NaL} density. Moreover, KN93 shifted the inactivation curves toward more depolarized potentials (from -80.5±1.3 to -73.2±2.4 mV in cells expressing Nav1.5 alone and from -82.5±2.0 to -73.5±2.6 mV in cells expressing Nav1.5+Kir2.1 channels; P<0.05) (Panel B). The results confirm the biophysical effects already attributed to the CaMKII-dependent phosphorylation of Nav1.5 channels. Interestingly, the I_{NaL} generated by Nav1.5 in the presence of Kir2.1 channels was significantly greater than that generated by Nav1.5 channels alone (P<0.05). These results would add further support to the hypothesis that CaMKII interacts with the Nav1.5-Kir2.1 complexes and is important for the positive reciprocal modulation to occur.

On the other hand, $I_{Kir2.1}$ and $I_{Nav1.5}$ were recorded in the presence of the AIP peptide in cells only transfected with Kir2.1 or Nav1.5 channels and in cells cotransfected with Nav1.5 and Kir2.1 channels. AIP was added at the "internal" solution that dialyzes the cells after the patch rupture. The tip of the pipette was always filled with AIP-free internal solution, in order to obtain the “control” current records. The results obtained (Panels C and D) confirmed those obtained with KN93, i.e., Kir2.1 channels are sensitive to CaMKII inhibition only when they are forming complexes with Nav1.5 channels.
