# Supplementary figures and images for: Kir2.1-Nav1.5 Channel Complexes Are Differently Regulated than Kir2.1 and Nav1.5 Channels Alone
Source: Front Physiol. 2017 Nov 14;8:903. doi: 10.3389/fphys.2017.00903 (PMC5694551; doi:10.3389/fphys.2017.00903)

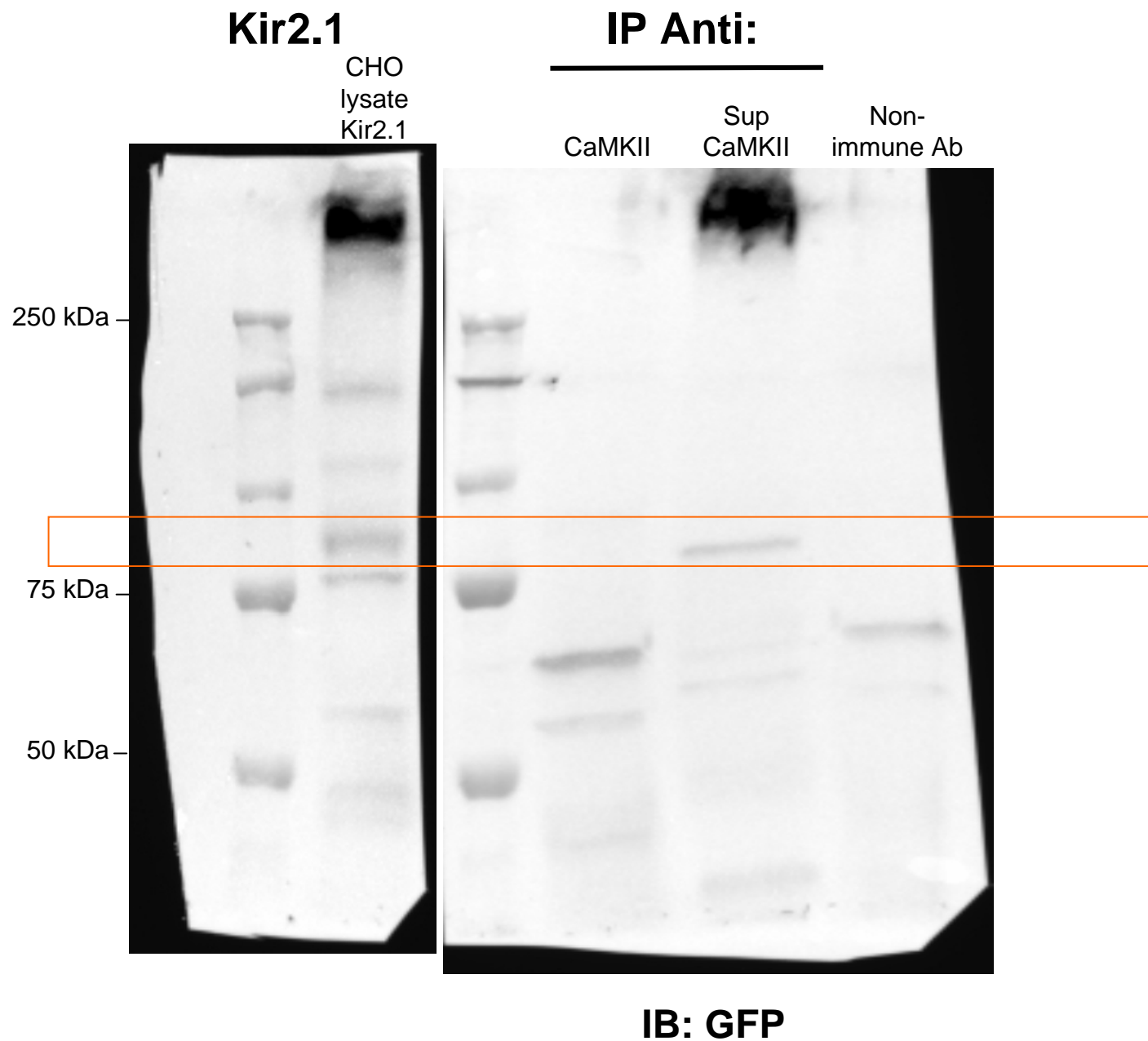

**Supplemental Figure 3.** Original blot of the image shown in Figure 3E.

Supplement: Supplementary file 3 [file Image3.PDF]

# Kir2.1+ Nav1.5

## IP Anti:

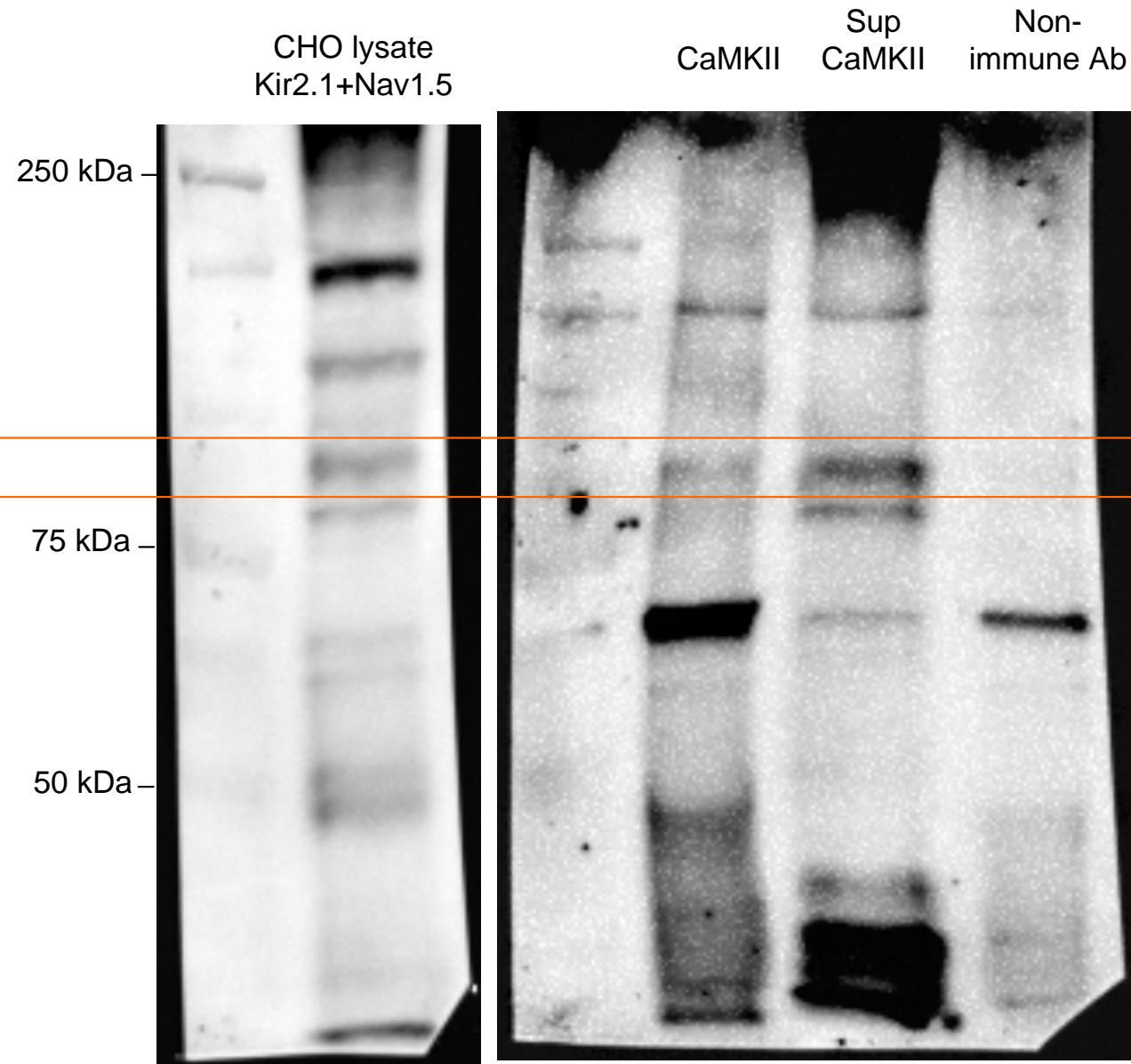

**IB: GFP**

**Supplemental Figure 4.** Original blot of the image shown in Figure 3F.

Supplement: Supplementary file 4 [file Image4.PDF]
