# Supplementary material for: Kir2.1-Nav1.5 Channel Complexes Are Differently Regulated than Kir2.1 and Nav1.5 Channels Alone
Source: Front Physiol. 2017 Nov 14;8:903. doi: 10.3389/fphys.2017.00903 (PMC5694551; doi:10.3389/fphys.2017.00903)
Supplement: Supplementary file 5 [file Image5.PDF]

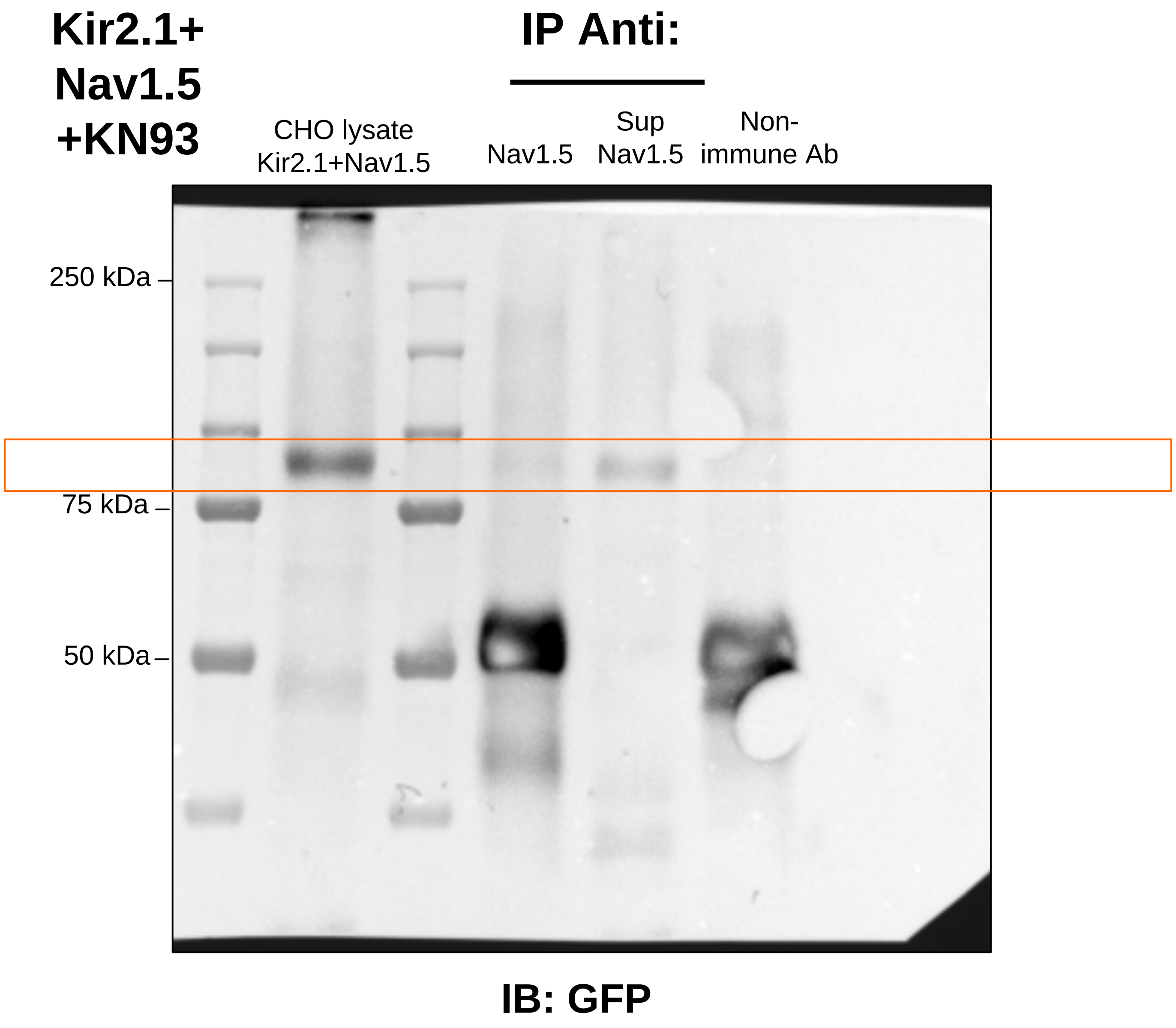

Supplemental Figure 5. Original blot of the image shown in Figure 3G.
